# Protocol for the insight study: a randomised controlled trial of single-dose tocilizumab in patients with depression and low-grade inflammation

**DOI:** 10.1136/bmjopen-2018-025333

**Published:** 2018-09-21

**Authors:** Golam M Khandaker, Bianca P Oltean, Muzaffer Kaser, Claire R M Dibben, Rajini Ramana, Deepak R Jadon, Robert Dantzer, Alasdair J Coles, Glyn Lewis, Peter B Jones

**Affiliations:** 1 Department of Psychiatry, University of Cambridge, Cambridge, UK; 2 Cambridgeshire and Peterborough NHS Foundation Trust, Fulbourn, UK; 3 Norfolk and Suffolk NHS Foundation Trust, Bury St Edmunds, UK; 4 Department of Rheumatology, Cambridge University Hospitals NHS Foundation Trust, Cambridge, UK; 5 Department of Symptom Research, MD Anderson Cancer Centre, University of Texas in Houston, Houston, Texas, USA; 6 Department of Clinical Neurosciences, University of Cambridge, Cambridge, UK; 7 Division of Psychiatry, University College London, London, UK

**Keywords:** clinical trials

## Abstract

**Introduction:**

Observational studies indicate a potentially causal role for interleukin 6 (IL-6), a proinflammatory cytokine, in pathogenesis of depression, but interventional studies based on patients with depression have not been conducted. Tocilizumab, anti-inflammatory drug, is a humanised monoclonal antibody that inhibits IL-6 signalling and is licensed in the UK for treatment of rheumatoid arthritis. The main objectives of this study are to test whether IL-6 contributes to the pathogenesis of depression and to examine potential mechanisms by which IL-6 affects mood and cognition. A secondary objective is to compare depressed participants with and without evidence of low-grade systemic inflammation.

**Methods and analysis:**

This is a proof-of-concept, randomised, parallel-group, double-blind, placebo-controlled clinical trial. Approximately 50 participants with International Classification of Diseases 10th revision (ICD-10) diagnosis of depression who have evidence of low-grade inflammation, defined as serum high-sensitivity C reactive protein (hs-CRP) level ≥3 mg/L, will receive either a single intravenous infusion of tocilizumab or normal saline. Blood samples, behavioural and cognitive measures will be collected at baseline and after infusion around day 7, 14 and 28. The primary outcome is somatic symptoms score around day 14 postinfusion. In addition, approximately, 50 depressed participants without low-grade inflammation (serum hs-CRP level <3 mg/L) will complete the same baseline assessments as the randomised cohort.

**Ethics and dissemination:**

The study has been approved by the South Central—Oxford B Research Ethics Committee (REC) (Reference: 18/SC/0118). Study findings will be published in peer-review journals. Findings will be also disseminated by conference/departmental presentations and by social and traditional media.

**Trial registration number:**

ISRCTN16942542; Pre-results.

**Strengths and limitations of this study**

- This is one of the first studies to examine the role of interleukin 6 in patients with depression using a randomised controlled trial design.
- The study examines important intermediate markers for antidepressant effect including somatic symptoms of depression, which will provide mechanistic insights into potential role of inflammation in depression.
- Comparison between inflamed and non-inflamed patients will help to better characterise patients with inflammation-related depression.
- As single dose of tocilizumab will be used, the study may not be able to test conclusively the efficacy of tocilizumab as potential treatment for depression.

## Introduction

### Scientific background and study rationale

Low-grade systemic inflammation as reflected by elevated concentrations of circulating inflammatory markers in peripheral blood may play a role in pathogenesis of depression.^1 2^ Meta-analysis of cross-sectional studies confirms that concentrations of circulating inflammatory cytokines, such as interleukin 6 (IL-6) and acute phase proteins, such as C reactive protein (CRP), are elevated in acutely unwell patients with depression,^3–5^ which tend to normalise after recovery^5^ but continue to be elevated in treatment resistant patients.^6 7^ Experimental studies indicate IL-6 and other cytokines are important mediators of the effects of inflammation on the brain.^1 2^ However, it is unclear whether inflammation plays a causal role in depression because cytokine elevation could be a consequence of depression (ie, reverse causality) or due to confounding. Existing epidemiological studies have addressed these issues to some extent (below), but randomised controlled trials (RCTs) of anticytokine treatment in depression are scarce. Intervention studies targeting the IL-6 system are required for a better understanding of the relationship between inflammation and depression.

**Figure 1:**
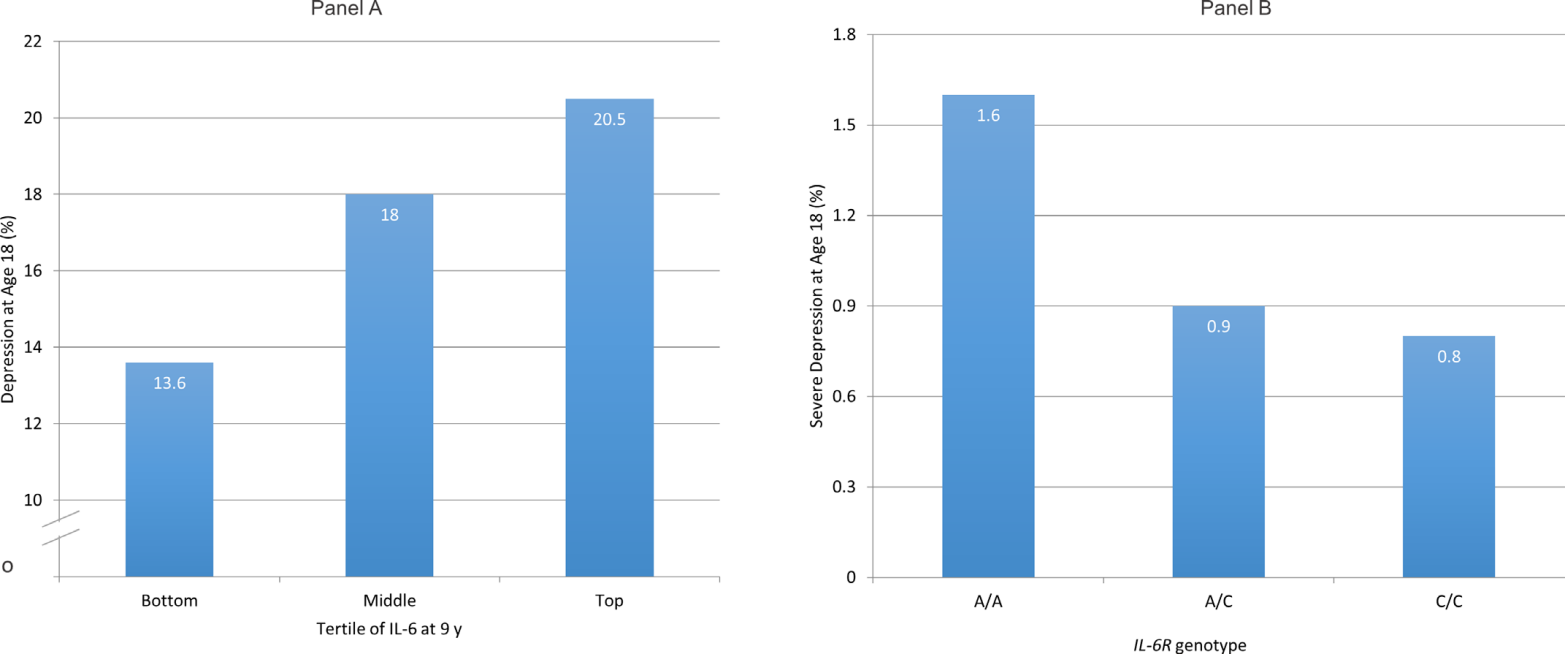
Cases of depression at age 18 in the ALSPAC birth cohort grouped by serum IL-6 levels at age 9 (A) and by *IL-6R* genotype (B). (A) Samples of depression were divided by tertiles of interleukin 6 (IL-6) in participants at age 9 years. Cut-off values for the top and bottom thirds of the distribution of IL-6 values in the total sample (cases and non-cases combined) were 1.08 and 0.57 pg/mL, respectively (reproduced with permission from Khandaker *et al*^9^). Panel (B) has been adapted from Khandaker *et al*.^10^ALSPAC, Avon Longitudinal Study of Parents and Children; IL-6R, interleukin 6 receptor.

Population-based longitudinal studies showing an association between elevated concentrations of IL-6 or CRP at baseline and increased risk of depression at follow-up indicates reverse causality is an unlikely explanation for previously observed association between IL-6 and depression.^8–11^ Using data from the Avon Longitudinal Study of Parents and Children (ALSPAC) birth cohort, we have reported that elevated concentrations of serum IL-6 in childhood are associated with increased risk of depression subsequently in early adulthood in a linear, dose–response fashion^9^ (figure 1A). Evidence for this association remains after controlling for potential confounders including sex, body mass index and maternal depression. This is one of the first evidence from humans that low-grade inflammation precedes depression, so is unlikely to be simply a consequence of illness.

Results from genetic association analysis informed by Mendelian randomisation (MR) indicate residual confounding is unlikely to explain the association between IL-6 and depression fully. MR is based on the idea that if a biomarker is causally related to an illness, genetic variant(s) regulating levels/activity of that biomarker should also be associated with the illness.^12 13^ Genetic variants segregate at random during meiosis and are unrelated to potential confounders, so using them as markers of exposure could overcome confounding. Using data from the ALSPAC birth cohort, we have shown that a genetic variant in the IL-6 receptor gene (*IL-6R* Asp358Ala; rs2228145) that is known to dampen down inflammation by impairing the activity of IL-6 is protective for severe depression^14^ (figure 1B). The genetic variant is strongly associated with serum IL-6 and CRP levels, but not with any common confounders of the inflammation–depression relationship such as sex, social class, ethnicity and body mass index. These findings strongly indicate that the IL-6/IL-6R pathways play a role in the pathophysiology of depression.

Although human population-based observational studies strongly support an association between IL-6 and depression, observational studies cannot confirm causality. RCTs are needed to test whether manipulation of IL-6 signalling has an impact on depressive symptoms in individuals with depression, but such studies are lacking. RCTs could also elucidate potential mechanisms by which IL-6 affects mood and cognition. Inflammation is unlikely to be relevant for all patients with depression,^15^ so consideration is required regarding the choice of suitable patients and outcomes for clinical trials of anti-inflammatory treatment to elucidate potential mechanistic role of the IL-6 system in depression (below).

### Stratified patient selection and choice of outcomes

A meta-analysis has reported that anticytokine drugs improve depressive symptoms in patients with chronic inflammatory illness, such as rheumatoid arthritis, independently of improvement in physical illness.^16^ Similarly, an RCT of infliximab (anti-Tumour Necrosis Factor alpha (TNF-α) monoclonal antibody and anti-inflammatory drug), which excluded patients with chronic physical illness, reported that the drug is more likely to improve depressive symptoms in patients with depression who show evidence of low-grade inflammation (ie, elevated CRP levels) at baseline.^17^ Therefore, clinical trials of IL-6 modulation should focus on depressed participants who have evidence of low-grade inflammation. Patients who do not get better with antidepressants are more likely to show evidence of low-grade inflammation.^6 7^

Inflammatory cytokines are more likely to be relevant for somatic symptoms of depression (eg, fatigue, appetite and sleep disturbance) rather than psychological symptoms (eg, hopelessness). Fatigue, sleep disturbance develop rapidly in majority of interferon-treated patients with cancer who develop depression (an established human model for inflammation-induced depression), but cognitive and affective symptoms (eg, impaired memory, low mood) develop slowly and relatively less frequently.^18 19^ Population-based studies have shown that elevated serum IL-6 and CRP levels are associated with fatigue, impaired sleep, but not with hopelessness.^20 21^ Cytokine-induced somatic symptoms may affect mood by reducing rewarding experiences,^22^ so could be a mediator of the relationship between inflammation and depression. This idea is consistent with our own work from the ALSPAC birth cohort which indicates that somatic symptoms mediate the association between IL-6 and psychological symptoms.^23^ Other groups have also reported that somatic symptoms of depression are associated with CRP, IL-6 and TNF-α levels.^24^ Therefore, somatic symptoms could be useful treatment target and marker of treatment response in clinical trials of anti-inflammatory treatment for depression. However, to our knowledge, no interventional study has examined the effects of reducing IL-6 activity on somatic symptoms specifically in individuals with depression.

Cognitive dysfunction, an unmet treatment need in depression,^25^ could be related to inflammation. Preclinical studies suggest that IL-6 may mediate inflammation-induced cognitive dysfunction in rats and mice.^26 27^ Neuroinflammation is associated with depressive symptoms and increased production of inflammatory cytokines in the hippocampus, a brain structure critical for memory.^28^ At population level, associations between circulating IL-6, CRP and general intelligence^29^ and cognitive symptoms of depression^8^ have been reported. In patients with depression, higher inflammatory marker levels are associated with poor psychomotor speed^30 31^ and persistent cognitive dysfunction.^32^ Therefore, in addition to depressive symptoms, inclusion of measures of cognitive function could provide useful insights into potential role of inflammation in depression.

### Proposed study

We propose a proof-of-concept, randomised, parallel-group, double-blind, placebo controlled clinical trial to investigate whether IL-6 contributes to pathogenesis of depression and to examine potential mechanisms by which IL-6 affects mood and cognition. We propose that inhibition of IL-6 signalling in individuals with depression who show evidence of low-grade inflammation and poor response to antidepressants would attenuate their depressive symptoms particularly somatic symptoms of depression.

Patients with depression who show evidence of inflammation may be different from those who do not. Those with low-grade inflammation are likely to be antidepressant resistant,^6 7^ so a clearer understanding of this group would be clinically useful. We also propose an observational study to examine similarities and differences between patients with depression with and without low-grade inflammation.

## Study aims and hypothesis

### Primary

To carry out a proof-of-concept, randomised, parallel-group, double-blind, placebo-controlled clinical trial to test whether IL-6 contributes to pathogenesis of depression and to examine potential mechanisms by which IL-6 affects mood and cognition. We hypothesise that inhibition of IL-6 signalling with a single intravenous infusion of anti-IL6R monoclonal antibody tocilizumab will attenuate somatic symptoms of depression, improve cognitive function, reduce serum proinflammatory cytokine levels and indoleamine 2,3-dioxygenase (IDO) activation in individuals with depression who show evidence of low-grade inflammation and poor response to antidepressant. Low-grade inflammation, hereafter also referred to as ‘inflamed depression’, will be defined as serum high-sensitivity CRP (hs-CRP) level ≥3 mg/L.

### Secondary

To carry out an observational study to examine differences and similarities between inflamed and non-inflamed depression (CRP <3 mg/L). We hypothesise that individuals with inflamed depression, compared with non-inflamed, will be more likely to have somatic symptoms, higher levels of serum proinflammatory cytokines, cognitive dysfunction and evidence of IDO activation.

## Methods

This protocol has been prepared in accordance with the Standard Protocol Items: Recommendations for Interventional Trials (SPIRIT) 2013 statement.^33^ Please see online supplementary eTable 1 for the SPIRIT checklist.

10.1136/bmjopen-2018-025333.supp1 Supplementary data

### Patient and public involvement

Patients were consulted during the development of the study protocol who contributed to the procedure, consent form and information leaflets. The study information was made more accessible and data collection assessments shorter. Interested participants will receive a summary of the main findings at the end of the study.

### Study design and sample

**Figure 2:**
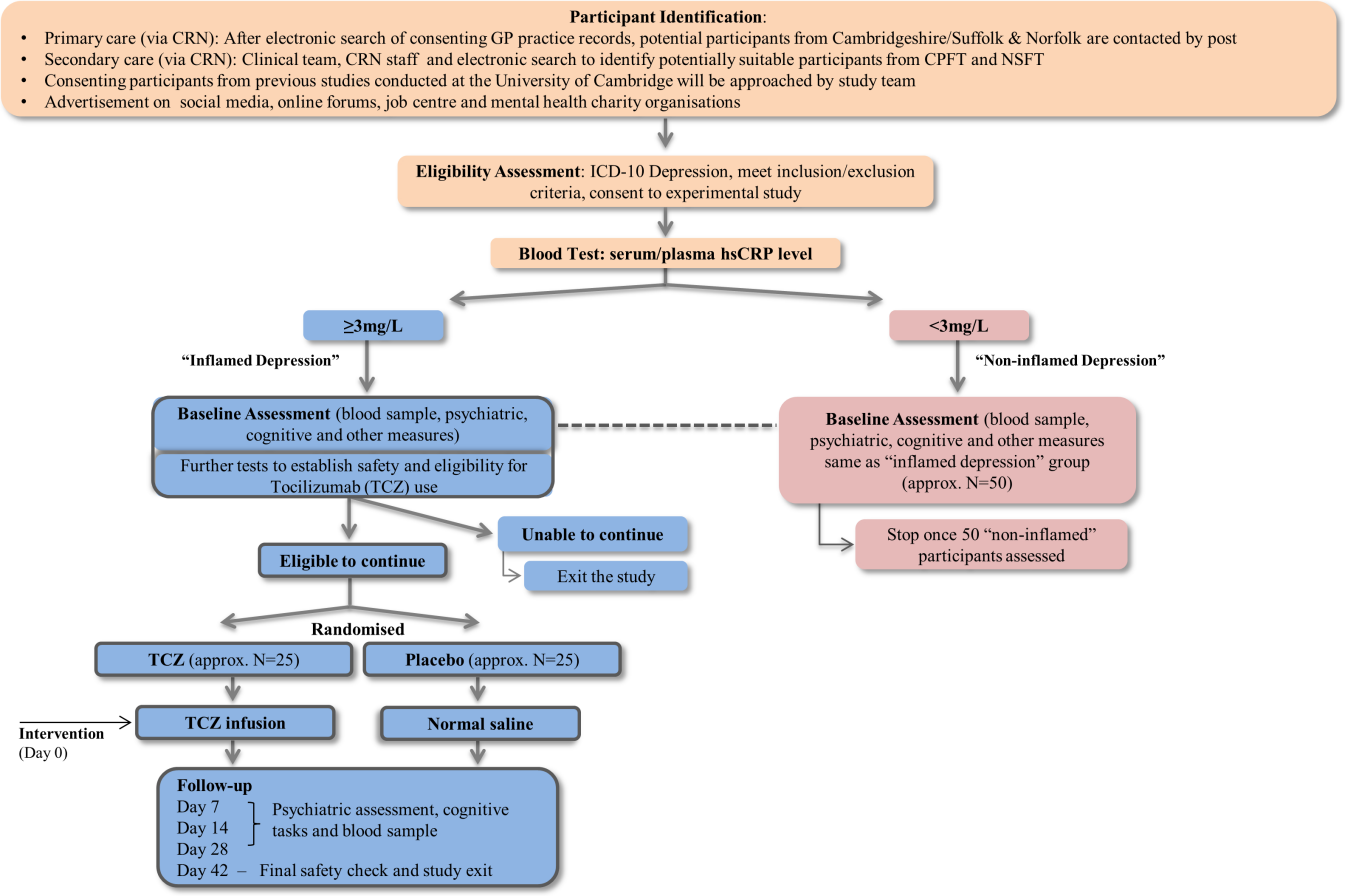
Overview of design and procedures for the insight study. CRN, clinical research network; CPFT, Cambridgeshire and Peterborough NHS Foundation Trust; GP, general practice; hs-CRP, high-sensitivity C reactive protein; NSFT, Norfolk and Suffolk NHS Foundation Trust.

The study has two parts. The clinical trial part will include approximately 50 patients with depression with CRP ≥3 mg/L (intervention cohort) who will be randomised into two groups (tocilizumab or normal saline). The observational part will compare the intervention cohort at baseline with 50 non-inflamed depressed patients (CRP <3 mg/L). Non-inflamed patients will not be randomised. For an overview of the study design, please see figure 2.

### Intervention

The study intervention will be a single intravenous infusion of tocilizumab (8 mg/kg; max 800 mg/patient) or normal saline. Tocilizumab is the first-in-class, anti-IL-6R humanised monoclonal antibody, which is commercially available and licensed in the UK for treating rheumatoid arthritis and juvenile idiopathic arthritis. Tocilizumab blocks both IL-6 classic and trans-signalling (responsible for most of the inflammatory effects of IL-6) making it the agent of choice for complete IL-6 inhibition.^34^ As justified by interferon^35^ and mouse^36^ studies, peripheral immune activation causes depression because IL-6 and other circulating cytokines can influence the brain using neural, humoral and cellular pathways.^1 2 37 38^ Therefore, tocilizumab which is mostly peripherally acting is likely to have an impact on symptoms of depression. Infliximab, an anti-TNF-α monoclonal antibody, that has similar, limited blood–brain barrier penetration as tocilizumab has been reported to modulate symptoms of depression.^17^ Similarly, peripherally acting anti-IL-6 monoclonal antibody has been reported to reduce depression-like behaviour in mice exposed to repeated stress.^39^

### Eligibility criteria

The study will include adult participants aged 20–65 years meeting ICD-10 criteria^40^ for depressive episode who are currently taking an antidepressant at adequate dose (according to British National Formulary or BNF) for at least 4 weeks. In addition, those included in the clinical trial part will have serum hs-CRP levels ≥3 mg/L. Please see table 1 for complete inclusion and exclusion criteria.

**Table 1:** Insight study inclusion and exclusion criteria

Group	Inclusion criteria	Exclusion criteria
All participants	Provide informed consent. Understand written and spoken English. Able to consent to blood sampling. Willing to abstain from strenuous exercise for 72 hours prior to assessment. Age: 20–65 years (inclusive). Diagnosis of depression: meet ICD-10 criteria at the time of assessment. Somatic symptom score: ≥7 at the time of eligibility based on Beck Depression Inventory-II items 4=lack of pleasure, 15=loss of energy, 16=changes in sleeping pattern, 18=changed in appetite, 19=concentration difficulty, 20=tiredness or fatigue and 21=loss of interest in sex. History of non/slow response to antidepressant: receiving treatment with an antidepressant at adequate dose (according to BNF) for at least 4 weeks.	Current or lifetime diagnosis of bipolar disorder, psychotic disorder, personality disorder or eating disorder. Current suicidal thoughts or history of suicide attempt, deliberate self-harm, overdose within 6 months prior to eligibility assessment. History of alcohol or substance use disorder (abuse/dependence) within 6 months prior to eligibility assessment. Pregnant or breast feeding. History of serious allergic reaction after any infusion. Current use of medication likely to compromise interpretation of immunological data (including, but not limited to, antibiotics, non-steroidal anti-inflammatory drugs, oral/injectable corticosteroids). Any major episode of infection requiring hospitalisation or treatment with intravenous antibiotics within 4 weeks of eligibility assessment. Presence or history of the following illnesses: recurrent bacterial, viral, fungal, mycobacterial or other opportunistic infections; unstable cardiac, pulmonary, renal, hepatic, endocrine, haematological or active infectious disease; rheumatic autoimmune disease, mixed connective tissue disease, scleroderma, polymyositis; uncontrolled hypertension. No history of chickenpox infection or no history of varicella zoster vaccination.
Intervention cohort	Serum/plasma high-sensitivity C reactive protein level ≥3 mg/L.	Current or past infection with tuberculosis (TB), hepatitis B, hepatitis C, HIV or Varicella Zoster Virus. History of severe allergic or anaphylactic reactions to human, humanised or murine monoclonal antibodies.

### Outcome

The primary outcome is change in total somatic symptom score from baseline to around day 14 postinfusion assessed using the Beck Depression Inventory-II^41^ (BDI-II). The somatic symptom score will be constructed by summing scores for seven relevant BDI-II items (4=lack of pleasure, 15=loss of energy, 16=changes in sleeping pattern, 18=changes in appetite, 19=concentration difficulty, 20=tiredness or fatigue and 21=loss of interest in sex). Depression severity is secondary outcome also assessed by BDI-II. As an experimental medicine study, we will collect data on a range of tertiary/exploratory measures including, but not limited to, fatigue, anhedonia, cognitive function and peripheral blood analyses of inflammatory markers, cortisol and markers of IDO activation.

### Statistical power

No RCTs were available to inform a power calculation for the proposed primary outcome. However, the clinical trial (n=50) will have 80% statistical power (alpha=0.05) to detect a 2.5-point reduction in Clinical Interview Schedule-Revised depression score in tocilizumab group compared with placebo; mean (SD) for outcome=15(3) based on a previous RCT of depression.^42^ We believe the actual sample size needed for the primary outcome will be smaller, as somatic symptoms are strongly influenced by inflammation.

### Randomisation and blinding

Participants will be randomly assigned to tocilizumab or normal saline group (1:1) using minimisation method to ensure that groups are comparable on somatic symptom severity and sex. Sealed Envelope, an external company, will do randomisation. Randomisation codes will be sent directly to the Central Pharmacy, Addenbrooke’s Hospital, Cambridge University Hospitals NHS Foundation Trust, who will dispense tocilizumab or normal saline according to randomisation schedule. Infusions will be prepared and administered at a clinical research facility (CRF) in Cambridge Biomedical Campus by CRF staff. Infusion packs containing drug or placebo will be visually indistinguishable ensuring both participants and study team remain blind about allocation of intervention.

### Statistical analysis

The clinical trial data will be analysed using an intention-to-treat approach. We will compare outcome measures between treatment and placebo groups controlling for baseline scores. This mechanistic experiment will focus on overall pattern of results rather than p values for individual tests of statistical significance. Analysis for the secondary observational study will compare psychiatric, cognitive and biomarkers between inflamed and non-inflamed groups using appropriate parametric and non-parametric statistical tests.

## Study procedure

### Identification of potentially eligible participants

An overview of study procedures has been presented in figure 2. The study has been adopted by the National Institute for Health Research (NIHR) clinical research network (CRN) portfolio. Recruitment sites will include Cambridgeshire and Peterborough NHS Foundation Trust (CPFT), Norfolk and Suffolk NHS Foundation Trust and primary care general practices (GPs) in Cambridgeshire and Suffolk. In addition, we will advertise in social media and in the community. Potentially eligible participants will be identified directly by clinicians, and by electronic search of clinical/research databases used by CPFT and GP surgeries. Participants will be first approached by their clinicians unless they had previously given consent to be contacted for research.

### Eligibility assessment

Face-to-face assessment will be carried out to establish eligibility and to obtain informed consent. A blood sample will be collected for serum hs-CRP measurement. In addition, participants identified from primary care will be first screened using a questionnaire containing key inclusion/exclusion criteria and the Patient Health Questionnaire-9.^43^

### Baseline data collection

All participants (50 inflamed and 50 non-inflamed) will attend a face-to-face assessment comprising psychiatric measures, cognitive tasks and blood sampling. Please see table 2 for a list of all study measures. This will be the final study contact for non-inflamed participants. Inflamed participants will undergo further tests to establish safety/eligibility to receive tocilizumab, which will include a chest X-ray and blood tests to exclude pregnancy and certain infections, such as TB, HIV. Eligible participants will be randomised and will be invited for infusion.

**Table 2:** Study measures

Domain	Tool	Source	Validated tool	Time of assessment
Sociodemographic/lifestyle	Screening questionnaire	Self-report		Screening
	Sociodemographic questionnaire	Self-report		Baseline
	Antidepressant history and concomitant treatment questionnaire	Self-report/general practice		Baseline
	Drug and alcohol questionnaire	Self-report		Baseline
Psychiatric	Patient Health Questionnaire-9	Self-report	✓	Screening
	Clinical Interview Schedule-Revised	Self-report	✓	Eligibility
	Beck Depression Inventory-II	Self-report	✓	Eligibility, baseline, follow-ups
	Snaith-Hamilton Pleasure Scale Questionnaire	Self-report	✓	Baseline, follow-ups
	Multidimensional Fatigue Inventory	Self-report	✓	Baseline, follow-ups
	Visual Analogue Scales for Subjective Feelings	Self-report		Baseline, follow-ups
	Perceived Stress Scale	Self-report	✓	Baseline
Cognitive	National Adult Reading Scale for estimated premorbid IQ	Interviewer assessed	✓	Baseline, follow-up 2
	Cambridge Neuropsychological Test Automated Battery (CANTAB) reaction time	Computer task	✓	Baseline, follow-up 2
	Symbol coding task	Computer/paper		Baseline, follow-up 2
	CANTAB Rapid Visual Information Processing	Computer task	✓	Baseline, follow-up 2
	CANTAB Paired Associates Learning	Computer task	✓	Baseline, follow-up 2
	Emotional categorisation and recall task	Computer task	✓	Baseline, follow-up 2
Biologic	Inflammatory markers, cardiometabolic markers, IDO activation, white cell phenotyping	Laboratory tests		Baseline, follow-ups
Genetic	Gene expression/genotyping	Blood (RNA, DNA)		Baseline, follow-up 2

### Intervention

Intravenous infusion of tocilizumab or normal saline will be given continuously over an hour at a CRF in Cambridge Biomedical Campus by trained clinical staff under the supervision of a designated study doctor. Participants will remain under clinical observation for a further 1-hour period after the end of infusion.

### Follow-up

Follow-up assessments will take place approximately 7, 14 and 28 days after infusion, and will be similar to baseline data collection. Cognitive tasks will be administered only on day 14. At around 42 days after infusion, participants will be contacted by phone to provide final debrief at which point they will exit the study.

## Risk management

### Depression-related risks

To minimise risk, all participants are required to be registered with a general practitioner, and give consent to access GP records to verify eligibility and to share clinically relevant findings with their GP. If a participant becomes distressed during an interview or does not wish to continue for any reason, the researcher will stop the interview and liaise with the study doctor to decide on appropriate course of action. Participants will be assessed for suicidality during eligibility assessment. Those with serious suicidal thoughts or history of suicide attempt, deliberate self-harm, overdose in 6 months prior to eligibility assessment will be excluded.

### Procedure-related risks

#### Venepuncture

The study requires blood draw. Blood taking is associated with mild discomfort and bruising though serious side effects are rare. Efforts will be made to minimise risk. Blood taking will be performed by a nurse, doctor or research team member trained in venepuncture.

#### Chest X-ray

Participants in the intervention cohort will receive a chest X-ray to screen for TB with a typical effective radiation dose of 0.016 mSv. This X-ray is additional to any standard clinical care outside of the trial. The dose is equivalent to that received, on average in the UK, from natural sources of radiation in the environment every 3–10 days. All examinations will need to be completed in compliance with local Ionising Radiation Medical Exposure Regulations Employer’s Procedures.

#### CRP levels

Participants will be informed of their serum hs-CRP level. The proposed threshold for defining participants as ‘inflamed’ used in this study is serum CRP level ≥3 mg/L. Having serum hs-CRP level above this threshold is not necessarily a cause for concern. About 30% of the general population have serum CRP levels 3.0–9.9 mg/L, and about 10%–15% have levels>10 mg/L.^44^ Reasons for elevated CRP in the absence of an infection or chronic inflammatory illness could include obesity, smoking, alcohol use, lack of exercise, so knowledge of CRP levels might prompt participants to adopt a healthier lifestyle. If serum CRP level is very high (>20 mg/L) without any apparent explanation such as infection or chronic inflammatory illness, we will inform the GP and the participant will be excluded from the study.

#### Risks to research staff

When home visits or lone working are required, staff will follow local safety procedures. Home visits will be conducted in pairs whenever possible for first visits.

### Safety considerations for infusion and monitoring of adverse reaction

#### Before infusion

Participants will be selected based on strict inclusion and exclusion criteria to minimise risk. In addition, we will carry out blood/other tests to exclude TB, HIV, VZV, hepatitis B and C because, though unlikely after a single dose, tocilizumab infusion could make these infections worse. Female participants of childbearing age will be given blood test for pregnancy. Participants who are sexually active will be asked to use contraception for 6 weeks after infusion. Male participants will be also asked not to donate sperm samples for 6 weeks after infusion.

#### During infusion

Infusions will be given under supervision of a designated study doctor. Participants will be monitored throughout the duration of infusion; vital signs and possible side effects will be recorded. Any immediate adverse events will be managed in line with the Addenbrooke’s Clinical Research Centre adverse event policy (ACRC/SOP018).

#### After infusion

Participants will remain under observation for a 1-hour period after infusion. Participants will be advised to seek help through GP or hospital accident and emergency (A&E) department if they feel unwell after leaving the hospital. Before leaving the CRF, participants will be given an information sheet containing a telephone number their health professionals can call. If necessary, we will unblind the participant and inform their health professional whether the participant had received tocilizumab or normal saline. Adverse reactions will be recorded at every follow-up and at final contact over the phone on day 42 after infusion. The expected time for complete elimination of tocilizumab from the system is approximately 42 days after a single infusion.^45^

## Ethics and dissemination

The study will be conducted in accordance with guidelines from the REC, Health Research Authority (HRA) and local Research and Development (R&D) departments. The study team will prepare protocol amendments as required and ethics approval will be sought before implementing any changes to the approved protocol. The ISRCTN Trial Registry and the Research Governance Office will be informed of any amendments to the protocol.

### Consent

Informed consent will be obtained for screening and for participation in the study. This will include consent to randomise, to contact GP to inform about participation in study, to access GP/psychiatric records to verify medical history to establish eligibility and to inform GP any results/outcomes as necessary. Consent for additional tests to establish safety for tocilizumab infusion and for storing biological samples will be also obtained. Participants have the option to opt in to being contacted for future studies.

### Study management

The Chief Investigator (CI) will have overall responsibility for the study. A named principal investigator (PI) will take clinical responsibility for the research team at each site. The CI will meet the study team regularly to discuss any issues. The study does not require the formal arrangement of a steering committee, because, according to HRA, it is not a Clinical Trial of an Investigational Medicinal Product. However, to enhance monitoring of study, a study management group has been set up, which includes academic and clinical experts in psychiatry, rheumatology, neuroscience and immunology.

### Data management and retention of samples

All potential participants will be assigned a unique study-specific participant ID number. All data, including personal details identifying individuals and anonymised data, will be subject to good practice as laid down in the Data Protection Act. Each stage of inviting, informing and assessing a particular participant is tracked so that their (anonymised) current status within the study is known and assessment and other appointment dates are forecasted. This information is held on the secure, password-protected database. Anonymised data from assessments will be uploaded to the secure, password-protected database using web-based data entry systems, transcribing from paper copy as necessary. Minimal personal data (eg, date of birth, sex) could be indexed by each participant’s unique ID number.

Blood samples collected in this study may be stored for up to 10 years after the completion of the study for additional research. Stored samples will be coded throughout the sample storage and analysis process and will not be labelled with personal identifiers. Participants may withdraw their consent for their samples to be stored for future research.

### Dissemination plan

Study findings will be published in peer-review journals. Publications will conform to the guidelines of the International Committee of Medical Journal Editors. Findings will be disseminated at conferences, departmental talks and other scientific meetings. Further dissemination will take place via social and traditional media.

## Supplementary Material

Reviewer comments

Author's manuscript
