# Supplementary material for: Protocol for the insight study: a randomised controlled trial of single-dose tocilizumab in patients with depression and low-grade inflammation
Source: BMJ Open. 2018 Sep 21;8(9):e025333. doi: 10.1136/bmjopen-2018-025333 (PMC6157523; doi:10.1136/bmjopen-2018-025333)
Supplement: Author's manuscript [file bmjopen-2018-025333.draft_revisions.pdf]

BMJ Open is committed to open peer review. As part of this commitment we make the peer review history of every article we publish publicly available.

When an article is published we post the peer reviewers' comments and the authors' responses online. We also post the versions of the paper that were used during peer review. These are the versions that the peer review comments apply to.

The versions of the paper that follow are the versions that were submitted during the peer review process. They are not the versions of record or the final published versions. They should not be cited or distributed as the published version of this manuscript.

BMJ Open is an open access journal and the full, final, typeset and author-corrected version of record of the manuscript is available on our site with no access controls, subscription charges or pay-per-view fees (http://bmjopen.bmj.com).

If you have any questions on BMJ Open's open peer review process please email editorial.bmjopen@bmj.com

BMJ Open

Protocol for the Insight Study: a randomised controlled trial of single dose tocilizumab in patients with depression and low-grade inflammation

Journal:	*BMJ Open*
Manuscript ID	bmjopen-2018-025333
Article Type:	Protocol
Date Submitted by the Author:	10-Jul-2018

Complete List of Authors:	Khandaker, Golam; University of Cambridge School of Clinical Medicine, Psychiatry<br>Oltean, Bianca; University of Cambridge School of Clinical Medicine, Psychiatry<br>Kaser, Muzaffer; University of Cambridge School of Clinical Medicine, Psychiatry<br>Dibben, Claire; Norfolk and Suffolk NHS Foundation Trust<br>Ramana, Rajini; Cambridgeshire and Peterborough NHS Foundation Trust<br>Jadon, Deepak; Cambridge University Hospitals NHS Foundation Trust<br>Dantzer , R ; Department of Symptom Research, MD Anderson Cancer Center, Houston<br>Coles, Alasdair; Department of Clinical Neurosciences, University of Cambridge<br>Lewis, Glyn; UCL Psychiatric Epidemiology, Mental Health Sciences Unit<br>Jones, Peter; University of Cambridge, Department of Psychiatry

Keywords:	Depression & mood disorders < PSYCHIATRY, Clinical trials < THERAPEUTICS, Protocols & guidelines < HEALTH SERVICES ADMINISTRATION & MANAGEMENT

SCHOLARONE™
Manuscripts

Protocol for the Insight Study: a randomised controlled trial of single dose tocilizumab in patients with depression and low-grade inflammation

Golam M Khandaker[1,2], Bianca P Oltean[1,2], Muzaffer Kaser[1,2], Claire R M Dibben[3], Rajini Ramana[1,2], Deepak R Jadon[4], Robert Dantzer[5], Alasdair Coles[6], Glyn Lewis[7], Peter B Jones[1,2]

[1] Department of Psychiatry, University of Cambridge, Cambridge, UK

[2] Cambridgeshire and Peterborough NHS Foundation Trust, Cambridge, UK

[3] Norfolk and Suffolk NHS Foundation Trust, Bury St Edmunds, UK

[4] Department of Rheumatology, Cambridge University Hospitals NHS Foundation Trust, Cambridge, UK

[5] Department of Symptom Research, MD Anderson Cancer Centre, University of Texas, Huston, USA

[6] Department of Clinical Neurosciences, University of Cambridge, Cambridge, UK

[7] Division of Psychiatry, University College London, London, UK

Correspondence to: Dr Golam Khandaker; Email: gmk24@medschl.cam.ac.uk

Word count:

Abstract: 259

Manuscript: 3445

Tables and Figures: 1 Table and 3 Figures (plus one online supplementary Table)

For peer review only

ABSTRACT

Introduction

Observational studies indicate a potentially causal role for interleukin 6 (IL-6), a proinflammatory cytokine, in pathogenesis of depression, but interventional studies based on patients with depression have not been conducted. Tocilizumab, anti-inflammatory drug, is a humanised monoclonal antibody that inhibits IL-6 signalling and is licensed in the UK for treatment of rheumatoid arthritis. The main objectives of this study are to test whether IL-6 contributes to the pathogenesis of depression, and to examine potential mechanisms by which IL-6 affects mood and cognition. A secondary objective is to compare depressed participants with and without evidence of low-grade systemic inflammation.

Methods and analysis

This is a proof-of-concept, randomised, parallel group, double blind, placebo-controlled clinical trial. Approximately 50 participants with ICD-10 diagnosis of depression who have evidence of low-grade inflammation, defined as serum high sensitivity C-reactive protein (hsCRP) level ≥3mg/L, will receive either a single intravenous infusion of tocilizumab or normal saline. Blood samples, behavioural and cognitive measures will be collected at baseline and after infusion around day 7, 14 and 28. The primary outcome is somatic symptoms score around day 14 post-infusion. In addition, approximately 50 depressed participants without low-grade inflammation (serum hsCRP level <3mg/L) will complete the same baseline assessments as the randomised cohort.

Ethics and dissemination

The study has been approved by the South Central - Oxford B Research Ethics Committee (Reference: 18/SC/0118). Study findings will be published in peer-review journals. Findings will be also disseminated by conference/departmental presentations, and by social and traditional media.

For peer review only - http://bmjopen.bmj.com/site/about/guidelines.xhtml

Trial registration number

ISRCTN16942542 (prospective registration pre first participant first visit).

Link: http://www.isrctn.com/ISRCTN16942542

Key words

Depression; Somatic Symptoms; Interleukin 6; Interleukin 6 Receptor; Tocilizumab; Monoclonal Antibody; Randomised Controlled Trial; Clinical Trial

For peer review only

For peer review only - http://bmjopen.bmj.com/site/about/guidelines.xhtml

Strengths and limitations of this study

- This is one of the first studies to examine the role of IL-6 in patients with depression using a randomised controlled trial design.
- The study examines important intermediate markers for antidepressant effect including somatic symptoms of depression, which will provide mechanistic insights into potential role of inflammation in depression.
- Comparison between inflamed and non-inflamed patients will help to better characterise patients with inflammation-related depression.
- As single dose of tocilizumab will be used, the study may not be able to test conclusively the efficacy of tocilizumab as potential treatment for depression.

For peer review only

INTRODUCTION

Scientific Background and Study Rationale

Low-grade systemic inflammation as reflected by elevated concentrations of circulating inflammatory markers in peripheral blood may play a role in pathogenesis of depression[1 2]. Meta-analysis of cross-sectional studies confirm that concentrations of circulating inflammatory cytokines, such as interleukin 6 (IL-6) and acute phase proteins, such as C-reactive protein (CRP), are elevated in acutely unwell patients with depression[3-5], which tend to normalise after recovery[5] but continue to be elevated in treatment resistant patients[6 7]. Experimental studies indicate IL-6 and other cytokines are important mediators of the effects of inflammation on the brain[1 2]. However, it is unclear whether inflammation plays a causal role in depression because cytokine elevation could be a consequence of depression (i.e. reverse causality) or due to confounding. Existing epidemiological studies have addressed these issues to some extent (below), but randomised controlled trials (RCT) of anti-cytokine treatment in depression are scarce. Intervention studies targeting the IL-6 system are required for a better understanding of the relationship between inflammation and depression.

Population-based longitudinal studies showing an association between elevated concentrations of IL-6 or CRP at baseline and increased risk of depression at follow-up indicates reverse causality is an unlikely explanation for previously observed association between IL-6 and depression [8-11]. Using data from the Avon Longitudinal Study of Parents and Children (ALSPAC) birth cohort, we have reported that elevated concentrations of serum IL-6 in childhood are associated with increased risk of depression subsequently in early-adulthood in a linear, dose-response fashion[9] (Figure 1, Panel A). Evidence for this association remains after controlling for potential confounders including sex, body mass index, and maternal depression. This is one of the first evidence from humans that low-grade inflammation precedes depression, so is unlikely to be simply a consequence of illness.

For peer review only - http://bmjopen.bmj.com/site/about/guidelines.xhtml

Figure 1: Cases of Depression at Age 18 in the ALSPAC Birth Cohort Grouped by Serum IL-6 Levels at Age 9 (Panel A) and by *IL-6R* Genotype (Panel B)

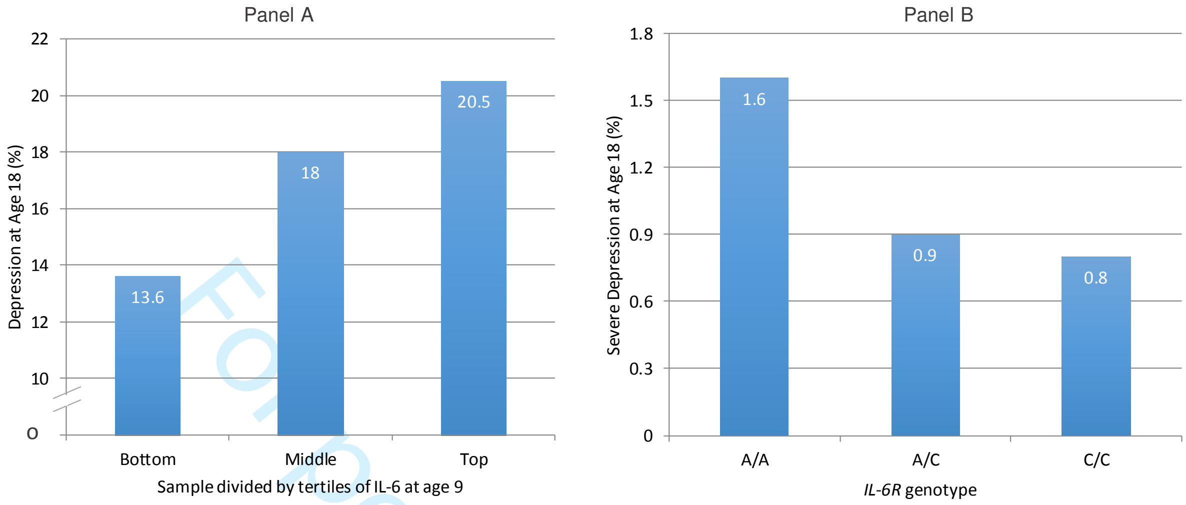

Note: Panel A was adapted from Khandaker *et al*. JAMA Psychiatry. 2014 Oct;71(10):1121-8; Panel B was adapted from Khandaker *et al*. Brain Behaviour Immunity. 2018 Mar;69:264-272

Results from genetic association analysis informed by Mendelian randomization (MR) indicate residual confounding is unlikely to explain the association between IL-6 and depression fully. MR is based on the idea that if a biomarker is causally related to an illness, genetic variant(s) regulating levels/activity of that biomarker should also be associated with the illness[12 13]. Genetic variants segregate at random during meiosis and are unrelated to potential confounders, so using them as markers of exposure could overcome confounding. Using data from the ALSPAC birth cohort, we have shown that a genetic variant in the IL-6 receptor gene (*IL6R* Asp358Ala; rs2228145) that is known to dampen down inflammation by impairing the activity of IL-6 is protective for severe depression[14] (Figure 1, Panel B). The genetic variant is strongly associated with serum IL-6 and CRP levels, but not with any common confounders of the inflammation-depression relationship such as sex, social class, ethnicity, and body mass index. These findings strongly indicate that the IL-6/IL-6R pathways play a role in the pathophysiology of depression.

For peer review only - http://bmjopen.bmj.com/site/about/guidelines.xhtml

Although human population-based observational studies strongly support an association between IL-6 and depression, observational studies cannot confirm causality. RCTs are needed to test whether manipulation of IL-6 signalling has an impact on depressive symptoms in individuals with depression, but such studies are lacking. RCTs could also elucidate potential mechanisms by which IL-6 affects mood and cognition. Inflammation is unlikely to be relevant for all patients with depression[15], so consideration is required regarding the choice of suitable patients and outcomes for clinical trials of anti-inflammatory treatment to elucidate potential mechanistic role of the IL-6 system in depression (below).

Stratified Patient Selection and Choice of Outcomes

A meta-analysis has reported that anti-cytokine drugs improve depressive symptoms in patients with chronic inflammatory illness, such as rheumatoid arthritis, independently of improvement in physical illness[16]. Similarly, an RCT of infliximab (anti-TNF monoclonal antibody and anti-inflammatory drug), which excluded patients with chronic physical illness, reported that the drug is more likely to improve depressive symptoms in depressed patients who show evidence of low-grade inflammation (i.e., elevated CRP levels) at baseline[17]. Therefore, clinical trials of IL-6 modulation should focus on depressed participants who have evidence of low-grade inflammation. Patients who do not get better with antidepressants are more likely to show evidence of low-grade inflammation[6 7].

Inflammatory cytokines are more likely to be relevant for somatic symptoms of depression (e.g. fatigue, appetite and sleep disturbance) rather than psychological symptoms (e.g. hopelessness). Fatigue, sleep disturbance develop rapidly in majority of interferon-treated cancer patients who develop depression (an established human model for inflammation-induced depression), but cognitive and affective symptoms (e.g. impaired memory, low mood) develop slowly and relatively less frequently[18 19]. Population-based studies have

For peer review only - http://bmjopen.bmj.com/site/about/guidelines.xhtml

shown that elevated serum IL-6 and CRP levels are associated with fatigue, impaired sleep, but not with hopelessness[20 21]. Cytokine-induced somatic symptoms may affect mood by reducing rewarding experiences[22], so could be a mediator of the relationship between inflammation and depression. This idea is consistent with our own work from the ALSPAC birth cohort which indicates that somatic symptoms mediate the association between IL-6 and psychological symptoms[23]. Other groups have also reported that somatic symptoms of depression are associated with CRP, IL-6, and TNF alpha levels[24]. Therefore, somatic symptoms could be useful treatment target and marker of treatment response in clinical trials of anti-inflammatory treatment for depression. However, to our knowledge no interventional study has examined the effects of reducing IL-6 activity on somatic symptoms specifically in individuals with depression.

Cognitive dysfunction, an unmet treatment need in depression[25], could be related to inflammation. Preclinical studies suggest that IL-6 may mediate inflammation-induced cognitive dysfunction in rats and mice[26 27]. Neuroinflammation is associated with depressive symptoms and increased production of inflammatory cytokines in the hippocampus, a bran structure critical for memory[28]. At population level, associations between circulating IL-6, CRP and general intelligence[29] and cognitive symptoms of depression[8] has been reported. In patients with depression, higher inflammatory marker levels are associated with poor psychomotor speed[30 31] and persistent cognitive dysfunction[32]. Therefore, in addition to depressive symptoms, inclusion of measures of cognitive function could provide useful insights into potential role of inflammation in depression.

For peer review only - http://bmjopen.bmj.com/site/about/guidelines.xhtml

Proposed Study

We propose a proof-of-concept, randomised, parallel group, double blind, placebo controlled clinical trial to investigate whether IL-6 contributes to pathogenesis of depression, and to examine potential mechanisms by which IL-6 affects mood and cognition. We propose that inhibition of IL-6 signalling in individuals with depression who show evidence of low-grade inflammation and poor response to antidepressants would attenuate their depressive symptoms particularly somatic symptoms of depression.

Patients with depression who show evidence of inflammation may be different from those who do not. Those with low-grade inflammation are likely to be antidepressant resistant[6 7], so a clearer understanding of this group would be clinically useful. We also propose an observational study to examine similarities and differences between depressed patients with and without low-grade inflammation.

STUDY AIMS AND HYPOTHESIS

Primary

To carry out a proof-of-concept, randomized, parallel group, double blind, placebo-controlled clinical trial to test whether IL-6 contributes to pathogenesis of depression, and to examine potential mechanisms by which IL-6 affects mood and cognition. We hypothesise that inhibition of IL-6 signalling with a single intravenous (IV) infusion of anti-IL6R monoclonal antibody tocilizumab will attenuate somatic symptoms of depression, improve cognitive function, reduce serum proinflammatory cytokine levels and IDO activation in individuals with depression who show evidence of low-grade inflammation and poor response to antidepressant. Low-grade inflammation, hereafter also referred to as ‘inflamed depression’, will be defined as serum high sensitivity CRP (hsCRP) level ≥3mg/L.

For peer review only - http://bmjopen.bmj.com/site/about/guidelines.xhtml

Secondary

To carry out an observational study to examine differences and similarities between inflamed and non-inflamed depression (CRP <3mg/L). We hypothesise that individuals with inflamed depression, compared with non-inflamed, will be more likely to have somatic symptoms, higher levels of serum proinflammatory cytokines, cognitive dysfunction and evidence of IDO activation.

METHODS

This protocol has been prepared in accordance with the Standard Protocol Items: Recommendations for Interventional Trials (SPIRIT) 2013 statement[33]. Please see eTable 1 for the SPIRIT checklist.

Study design and Sample

The study has two parts. The clinical trial part will include approximately 50 depressed patients with CRP ≥3mg/L (intervention cohort) who will be randomised into two groups (tocilizumab or normal saline). The observational part will compare the intervention cohort at baseline with 50 non-inflamed depressed patients (CRP <3mg/L). Non-inflamed patients will not be randomised. For an overview of the study design please see Figure 2.

For peer review only

Figure 2: Overview of Design and Procedures for the Insight Study

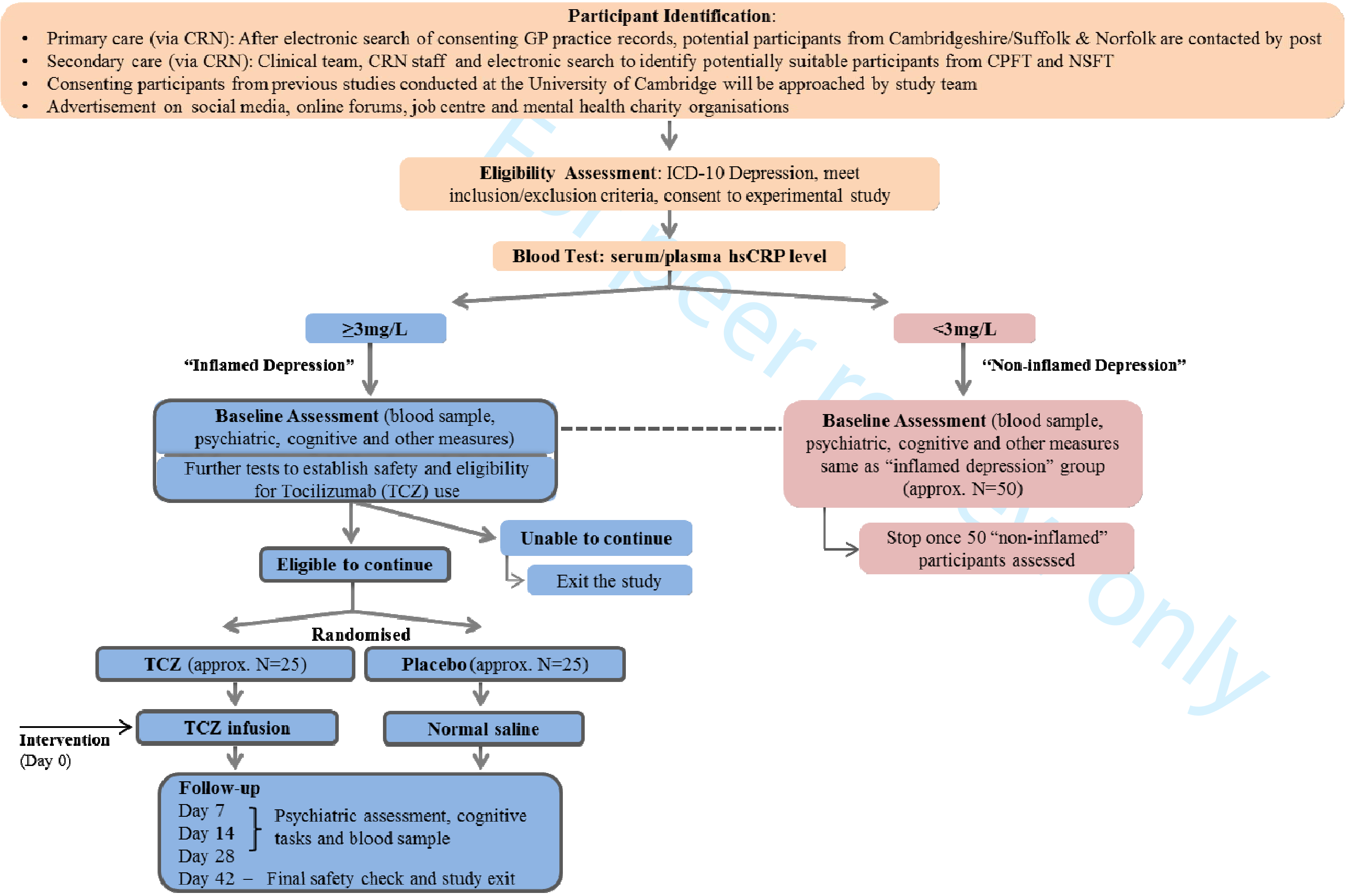

For peer review only - http://bmjopen.bmj.com/site/about/guidelines.xhtml

Intervention

The study intervention will be a single IV infusion of tocilizumab (8mgs/kg; max 800mgs/patient) or normal saline. Tocilizumab is the first-in-class, anti-IL-6R humanized monoclonal antibody, which is commercially available and licensed in the UK for treating rheumatoid arthritis and juvenile idiopathic arthritis. Tocilizumab blocks both IL-6 classic and trans-signaling (responsible for most of the inflammatory effects of IL-6) making it the agent of choice for complete IL-6 inhibition[34]. As justified by interferon[35] and mouse[36] studies, peripheral immune-activation causes depression because IL-6 and other circulating cytokines can influence the brain using neural, humoral and cellular pathways[1 2 37 38]. Therefore, tocilizumab which is mostly peripherally acting is likely to have an impact on symptoms of depression. Infliximab, an anti-TNF-α monoclonal antibody, that has similar, limited blood-brain barrier penetration as tocilizumab has been reported to modulate symptoms of depression[17]. Similarly, peripherally acting anti-IL-6 monoclonal antibody has been reported to reduce depression-like behaviour in mice exposed to repeated stress[39].

Eligibility criteria

The study will include adult participants aged 20 to 65 years meeting ICD-10 criteria[40] for depressive episode who are currently taking an antidepressant at adequate dose (according to BNF) for at least four weeks. In addition, those included in the clinical trial part will have serum hsCRP levels ≥3mg/L. Please see Figure 3 for complete inclusion and exclusion criteria.

For peer review only - http://bmjopen.bmj.com/site/about/guidelines.xhtml

Figure 3: Insight Study Inclusion and Exclusion Criteria

Eligibility Criteria		
**Group**	**Inclusion criteria**	**Exclusion criteria**
**All participants**	▪ Provide informed consent<br>▪ Understand written and spoken English<br>▪ Able to consent to blood sampling<br>▪ Willing to abstain from strenuous exercise for 72 hours prior to assessment<br>▪ **Age:** 20-65 years (inclusive)<br>▪ **Diagnosis of depression**: meet ICD-10 criteria at the time of assessment<br>▪ **Somatic symptom score**: ≥7 at the time of eligibility assessment based on Beck Depression Inventory II (BDI II) items 4=lack of pleasure, 15=loss of energy, 16=changes in sleeping pattern, 18=changes in appetite, 19=concentration difficulty, 20=tiredness or fatigue, and 21=loss of interest in sex<br>▪ **History of non/slow response to antidepressant:** receiving treatment with an antidepressant at adequate dose (according to BNF) for at least four weeks	▪ Current or lifetime diagnosis of bipolar disorder, psychotic disorder, personality disorder or eating disorder<br>▪ Current suicidal thoughts or history of suicide attempt, deliberate self-harm, overdose within six months prior to eligibility assessment<br>▪ History of alcohol or substance use disorder (abuse/dependence) within six months prior to eligibility assessment<br>▪ Pregnant or breast feeding<br>▪ History of serious allergic reaction after any infusion<br>▪ Current use of medication likely to compromise interpretation of immunological data (including, but not limited to, antibiotics, non-steroidal anti-inflammatory drugs, oral/injectable corticosteroids)<br>▪ Any major episode of infection requiring hospitalization or treatment with IV antibiotics within 4 weeks of eligibility assessment<br>▪ Presence or history of the following illnesses: recurrent bacterial, viral, fungal, mycobacterial or other opportunistic infections; unstable cardiac, pulmonary, renal, hepatic, endocrine, hematologic, or active infectious disease; rheumatic autoimmune disease, mixed connective tissue disease, scleroderma, polymyositis; uncontrolled hypertension<br>▪ No history of chicken pox infection or no history of varicella zoster vaccination
**Intervention Cohort**	▪ Serum/plasma hsCRP level ≥3mg/L	▪ Current or past infection with TB, Hepatitis B, Hepatitis C, HIV or VZV<br>▪ History of severe allergic or anaphylactic reactions to human, humanized or murine monoclonal antibodies

Outcome

The primary outcome is total somatic symptom score at around day 14 post-infusion assessed using the Beck Depression Inventor-II[41] (BDI-II). The somatic symptom score will be constructed by summing scores for seven relevant BDI-II items (4=lack of pleasure, 15=loss of energy, 16=changes in sleeping pattern, 18=changes in appetite, 19=concentration difficulty, 20=tiredness or fatigue, and 21=loss of interest in sex). We will collect data on range of secondary/exploratory outcomes including, but not limited to, depression severity, fatigue and anhedonia, cognitive function, and peripheral blood analyses of inflammatory markers, cortisol and markers of IDO activation.

For peer review only - http://bmjopen.bmj.com/site/about/guidelines.xhtml

Statistical power

No RCTs were available to inform a power calculation for the proposed primary outcome. However, the clinical trial (N=50) will have 80% statistical power (alpha=0.05) to detect a 2.5-point reduction in clinical interview schedule revised (CIS-R) depression score in tocilizumab group compared with placebo; mean (SD) for outcome=15(3) based on a previous RCT of depression[42]. We believe the actual sample size needed for the primary outcome will be smaller, as somatic symptoms are strongly influenced by inflammation.

Randomisation and blinding

Participants will be randomly assigned to tocilizumab or normal saline group (1:1) using minimization method to ensure that groups are comparable on somatic symptom severity and sex. Sealed Envelope, an external company, will do randomization. Randomization codes will be sent directly to the Central Pharmacy, Addenbrooke's Hospital, Cambridge University Hospitals NHS Foundation Trust, who will dispense tocilizumab or normal saline according to randomization schedule. Infusions will be prepared and administered at a clinical research facility (CRF) in Cambridge Biomedical Campus by CRF staff. Infusion packs containing drug or placebo will be visually indistinguishable ensuring both participants and study team remain blind about allocation of intervention.

Statistical analysis

The clinical trial data will be analysed using an intention-to-treat approach. We will compare outcome measures between treatment and placebo groups controlling for baseline scores. This mechanistic experiment will focus on overall pattern of results rather than *P*-values for individual tests of statistical significance. Analysis for the secondary observational study will compare psychiatric, cognitive, and biomarkers between inflamed and non-inflamed groups using appropriate parametric and non-parametric statistical tests.

For peer review only - http://bmjopen.bmj.com/site/about/guidelines.xhtml

STUDY PROCEDURE

Identification of potentially eligible participants

An overview of study procedures has been presented in Figure 2. The study has been adopted by the National Institute for Health Research (NIHR) clinical research network (CRN) portfolio. Recruitment sites will include Cambridgeshire and Peterborough NHS Foundation Trust (CPFT), Norfolk and Suffolk NHS Foundation Trust (NSFT), and primary care general practices (GP) in Cambridgeshire and Suffolk. In addition, we will advertise in social media and in the community. Potentially eligible participants will be identified directly by clinicians, and by electronic search of clinical/research databases used by CPFT and GP surgeries. Participants will be first approached by their clinicians unless they had previously given consent to be contacted for research.

Eligibility assessment

Face-to-face assessment will be carried out to establish eligibility and to obtain informed consent. A blood sample will collected for serum hsCRP measurement. In addition, participants identified from primary care will be first screened using a questionnaire containing key inclusion/exclusion criteria and the patient health questionnaire 9 (PHQ-9)[43].

Baseline data collection

All participants (50 inflamed and 50 non-inflamed) will attend a face-to-face assessment comprising psychiatric measures, cognitive tasks and blood sampling. Please see Table 1 for a list of all study measures. This will be the final study contact for non-inflamed participants. Inflamed participants will undergo further tests to establish safety/eligibility to receive tocilizumab, which will include a chest X-ray and blood tests to exclude pregnancy and certain infections, such as TB, HIV. Eligible participants will be randomised and will be invited for infusion.

For peer review only - http://bmjopen.bmj.com/site/about/guidelines.xhtml

Table 1: Study Measures

Domain	Tool	Source	Validated tool	Time of assessment
**Socio-demographic/ lifestyle**	Screening questionnaire	Self-report		Screening
	Sociodemographic questionnaire	Self-report		Baseline
	Antidepressant history & concomitant treatment questionnaire	Self-report/GP		Baseline
	Drug and alcohol questionnaire	Self-report		Baseline
**Psychiatric**	Patient Health Questionnaire 9	Self-report	✓	Screening
	Clinical Interview Schedule-Revised	Self-report	✓	Eligibility
	Beck Depression Inventory-II	Self-report	✓	Eligibility, Baseline, Follow-ups
	Snaith-Hamilton Pleasure Scale Questionnaire	Self-report	✓	Baseline, Follow-ups
	Multi-dimensional Fatigue Inventory	Self-report	✓	Baseline, Follow-ups
	Visual Analogue Scales for Subjective Feelings	Self-report		Baseline, Follow-ups
	Perceived Stress Scale	Self-report	✓	Baseline
**Cognitive**	National Adult Reading Scale for estimated premorbid IQ	Interviewer-assessed	✓	Baseline, Follow-up 2
	CANTAB Reaction Time	Computer task	✓	Baseline, Follow-up 2
	Symbol Coding Task	Computer / Paper		Baseline, Follow-up 2
	CANTAB Rapid Visual Information Processing	Computer task	✓	Baseline, Follow-up 2
	CANTAB Paired Associates Learning	Computer task	✓	Baseline, Follow-up 2
	Emotional categorization and recall task	Computer task	✓	Baseline, Follow-up 2
**Biologic**	Inflammatory markers, cardio-metabolic markers, IDO activation, White Cell phenotyping	Laboratory tests		Baseline, Follow-ups
**Genetic**	Gene expression/ Genotyping	Blood (RNA, DNA)		Baseline, Follow-up 2

Intervention

For peer review only - http://bmjopen.bmj.com/site/about/guidelines.xhtml

Intravenous infusion of tocilizumab or normal saline will be given continuously over an hour at a CRF in Cambridge Biomedical Campus by trained clinical staff under the supervision of a designated study doctor. Participants will remain under clinical observation for a further one-hour period after the end of infusion.

Follow-up

Follow-up assessments will take place approximately 7, 14 and 28 days after infusion, and will be similar to baseline data collection. Cognitive tasks will be administered only on day 14. At around 42 days after infusion, participants will be contacted by phone to provide final debrief at which point they will exit the study.

RISK MANAGEMENT

Depression-related Risks

To minimise risk, all participants are required to be registered with a general practitioner, and give consent to access GP records to verify eligibility and to share clinically relevant findings with their GP. If a participant becomes distressed during an interview, or does not wish to continue for any reason, the researcher will stop the interview and liaise with the study doctor to decide on appropriate course of action. Participants will be assessed for suicidality during eligibility assessment. Those with serious suicidal thoughts or history of suicide attempt, deliberate self-harm, overdose in six months prior to eligibility assessment will be excluded.

Procedure-related risks

For peer review only - http://bmjopen.bmj.com/site/about/guidelines.xhtml

Venepuncture

The study requires blood draw. Blood taking is associated with mild discomfort and bruising though serious side effects are rare. Efforts will be made to minimise risk. Blood taking will be performed by a nurse, doctor or research team member trained in venepuncture.

Chest X-ray

Participants in the intervention cohort will receive a chest x-ray to screen for Tuberculosis with a typical effective radiation dose of 0.016 mSv. This x-ray is additional to any standard clinical care outside of the trial. The dose is equivalent to that received, on average in the UK, from natural sources of radiation in the environment every three to ten days. All examinations will need to be completed in compliance with local Ionising Radiation Medical Exposure Regulations Employer's Procedures.

CRP Levels

Participants will be informed of their serum hsCRP level. The proposed threshold for defining participants as 'inflamed' used in this study is serum CRP level ≥3mg/L. Having serum hsCRP level above this threshold is not necessarily a cause for concern. About 30% of the general population have serum CRP levels 3.0-9.9mg/L, and about 10-15% have levels >10mg/L[44]. Reasons for elevated CRP in the absence of an infection or chronic inflammatory illness could include obesity, smoking, alcohol use, lack of exercise, so knowledge of CRP levels might prompt participants to adopt a healthier lifestyle. If serum CRP level is very high (>20mg/L) without any apparent explanation such as infection or chronic inflammatory illness, we will inform the GP and the participant will be excluded from the study.

Risks to research staff

For peer review only - http://bmjopen.bmj.com/site/about/guidelines.xhtml

When home visits or lone working are required, staff will follow local safety procedures. Home visits will be conducted in pairs whenever possible for first visits.

Safety considerations for infusion and monitoring of adverse reaction

Before infusion

Participants will be selected based on strict inclusion and exclusion criteria to minimise risk. In addition, we will carry out blood/other tests to exclude TB, HIV, VZV, Hepatitis B and C because, though unlikely after a single dose, tocilizumab infusion could make these infections worse. Female participants of childbearing age will be given blood test for pregnancy. Participants who are sexually active will be asked to use contraception for six weeks after infusion. Male participants will be also asked not to donate sperm samples for six weeks after infusion.

During infusion

Infusions will be given under supervision of a designated study doctor. Participants will be monitored throughout the duration of infusion; vital signs and possible side effects will be recorded. Any immediate adverse events will be managed in line with the Addenbrooke's Clinical Research Centre adverse event policy (ACRC/SOP018).

After infusion

Participants will remain under observation for a one-hour period after infusion. Participants will be advised to seek help through GP or A&E if they feel unwell after leaving the hospital. Before leaving the CRF, participants will be given an information sheet containing a telephone number their health professionals can call. If necessary we will un-blind the participant and inform their health professional whether the participant had received tocilizumab or normal saline. Adverse reactions will be recorded at every follow-up and at

For peer review only - http://bmjopen.bmj.com/site/about/guidelines.xhtml

final contact over the phone on day 42 after infusion. The expected time for complete elimination of tocilizumab from the system is approximately 42 days after a single infusion[45].

ETHICS AND DISSEMINATION

The study has been approved by the South Central - Oxford B Research Ethics Committee (REC) on 24/04/2018 (Reference: 18/SC/0118), and Health Research Authority (HRA) on 02/05/2018 (IRAS ID: 238297). The study will be conducted in accordance with guidelines from the REC, HRA and local R&D departments. The study team will prepare protocol amendments as required and ethics approval will be sought before implementing any changes to the approved protocol. The ISRCTN Trial Registry and the Research Governance Office will be informed of any amendments to the protocol.

Consent

Informed consent will be obtained for screening and for participation in the study. This will include consent to randomise, to contact GP to inform about participation in study, to access GP/psychiatric records to verify medical history to establish eligibility, and to inform GP any results/outcomes as necessary. Consent for additional tests to establish safety for tocilizumab infusion and for storing biological samples will be also obtained. Participants have the option to opt in to being contacted for future studies.

Study management

The CI will have overall responsibility for the study. A named principal investigator (PI) will take clinical responsibility for the research team at each site. The CI will meet the study team regularly to discuss any issues. The study does not require the formal arrangement of a steering committee, because, according to HRA, it is not a Clinical Trial of an Investigational Medicinal Product (CTIMP). However, to enhance monitoring of study, a Study Management

For peer review only - http://bmjopen.bmj.com/site/about/guidelines.xhtml

Group has been set up, which includes academic and clinical experts in psychiatry, rheumatology, neuroscience and immunology.

Data management and retention of samples

All potential participants will be assigned a unique study specific participant ID number. All data including personal details identifying individuals and anonymised data will be subject to good practice as laid down in the Data Protection Act. Each stage of inviting, informing and assessing a particular participant is tracked so that their (anonymised) current status within the study is known and assessment and other appointment dates are forecasted. This information is held on the secure, password protected database. Anonymised data from assessments will be uploaded to the secure, password protected database using web-based data entry systems, transcribing from paper copy as necessary. Minimal personal data (e.g., date of birth, sex) could be indexed by each participant's unique ID number.

Blood samples collected in this study may be stored for up to 10 years after the completion of the study for additional research. Stored samples will be coded throughout the sample storage and analysis process and will not be labelled with personal identifiers. Participants may withdraw their consent for their samples to be stored for research.

For peer review only

Dissemination plan

Study findings will be published in peer-review journals. Publications will conform to the guidelines of the International Committee of Medical Journal Editors (ICMJE). Interested participants will receive a summary of the main findings at the end of the study. Findings will be disseminated at conferences, departmental talks and other scientific meetings. Further dissemination will take place via social and traditional media.

Acknowledgements

For peer review only - http://bmjopen.bmj.com/site/about/guidelines.xhtml

The authors would like to thank NIHR CRN Eastern mental health and primary care teams for their help with preparing study protocol and materials. We are grateful to Ruth Holmes and Clare Knight from CRN for organising PPI reviews of a previous version of the study protocol, and respective PPI members for their useful comments and suggestions. We are grateful to Jane Gaffa from CPFT R&D dept. for her help with study approvals.

Contributors

GMK designed the study, obtained funding, developed protocol and study materials, obtained necessary approvals for the study, and revised this manuscript. BPO contributed to study design, developed protocol and study materials, obtained necessary approvals for the study, and wrote first draft for this manuscript. PBJ contributed to study design, obtaining funding, development of the protocol and revised this manuscript. AC, DRJ, GL, MK RD and RR contributed to study design, protocol and revised this manuscript. Coles, Dantzer, Jadon, Jones, Khandaker, Lewis, and Ramana are part of the Study Management Group. GMK is the chief investigator, lead researcher and guarantor of the study.s

Funding

The study is funded by Wellcome Trust Intermediate Clinical Fellowship to Khandaker (01486/Z/16/Z).

Study Sponsor

The study is jointly sponsored by the Cambridgeshire and Peterborough NHS Foundation Trust and the University of Cambridge.

Competing interests

For peer review only - http://bmjopen.bmj.com/site/about/guidelines.xhtml

The authors have no conflicts of interest to declare in relation to this study. The sponsors and funders had no role in the study design or any other aspects relating to the conduct or reporting of the study.

Patient consent

Informed consent will be obtained (see manuscript).

Ethics approval

The study has been approved by the South Central - Oxford B Research Ethics Committee (REC) on 24/04/2018 (Reference: 18/SC/0118).

For peer review only

For peer review only - http://bmjopen.bmj.com/site/about/guidelines.xhtml

Reference

1. Dantzer R, O'Connor JC, Freund GG, Johnson RW, Kelley KW. From inflammation to sickness and depression: when the immune system subjugates the brain. *Nature reviews. Neuroscience* 2008;9(1):46-56.

2. Miller AH, Maletic V, Raison CL. Inflammation and its discontents: the role of cytokines in the pathophysiology of major depression. *Biological psychiatry* 2009;65(9):732-41.

3. Howren MB, Lamkin DM, Suls J. Associations of depression with C-reactive protein, IL-1, and IL-6: a meta-analysis. *Psychosomatic medicine* 2009;71(2):171-86.

4. Haapakoski R, Mathieu J, Ebmeier KP, Alenius H, Kivimaki M. Cumulative meta-analysis of interleukins 6 and 1beta, tumour necrosis factor alpha and C-reactive protein in patients with major depressive disorder. *Brain, behavior, and immunity* 2015;49:206-15.

5. Goldsmith DR, Rapaport MH, Miller BJ. A meta-analysis of blood cytokine network alterations in psychiatric patients: comparisons between schizophrenia, bipolar disorder and depression. *Molecular psychiatry* 2016.

6. Maes M, Bosmans E, De Jongh R, Kenis G, Vandoolaeghe E, Neels H. Increased serum IL-6 and IL-1 receptor antagonist concentrations in major depression and treatment resistant depression. *Cytokine* 1997;9(11):853-8.

7. O'Brien SM, Scully P, Fitzgerald P, Scott LV, Dinan TG. Plasma cytokine profiles in depressed patients who fail to respond to selective serotonin reuptake inhibitor therapy. *Journal of psychiatric research* 2007;41(3-4):326-31.

8. Gimeno D, Kivimaki M, Brunner EJ, Elovainio M, De Vogli R, Steptoe A, et al. Associations of C-reactive protein and interleukin-6 with cognitive symptoms of depression: 12-year follow-up of the Whitehall II study. *Psychological medicine* 2009;39(3):413-23.

For peer review only - http://bmjopen.bmj.com/site/about/guidelines.xhtml

9. Khandaker GM, Pearson RM, Zammit S, Lewis G, Jones PB. Association of serum interleukin 6 and C-reactive protein in childhood with depression and psychosis in young adult life: a population-based longitudinal study. *JAMA psychiatry* 2014;71(10):1121-8.

10. Khandaker GM, Stochl J, Zammit S, Goodyer I, Lewis G, Jones PB. Childhood inflammatory markers and intelligence as predictors of subsequent persistent depressive symptoms: a longitudinal cohort study. *Psychological medicine* 2017:1-12.

11. Zalli A, Jovanova O, Hoogendijk WJ, Tiemeier H, Carvalho LA. Low-grade inflammation predicts persistence of depressive symptoms. *Psychopharmacology* 2016;233(9):1669-78.

12. Davey Smith G, Ebrahim S. 'Mendelian randomization': can genetic epidemiology contribute to understanding environmental determinants of disease? *International journal of epidemiology* 2003;32(1):1-22.

13. Burgess S, Timpson NJ, Ebrahim S, Davey Smith G. Mendelian randomization: where are we now and where are we going? *International journal of epidemiology* 2015;44(2):379-88.

14. Khandaker GM, Zammit S, Burgess S, Lewis G, Jones PB. Association between a functional interleukin 6 receptor genetic variant and risk of depression and psychosis in a population-based birth cohort. *Brain, behavior, and immunity* 2017.

15. Khandaker GM, Dantzer R, Jones PB. Immunopsychiatry: important facts. *Psychological medicine* 2017:1-9.

16. Kappelmann N, Lewis G, Dantzer R, Jones PB, Khandaker GM. Antidepressant activity of anti-cytokine treatment: a systematic review and meta-analysis of clinical trials of chronic inflammatory conditions. *Molecular psychiatry* 2018;23(2):335-43.

17. Raison CL, Rutherford RE, Woolwine BJ, Shuo C, Schettler P, Drake DF, et al. A randomized controlled trial of the tumor necrosis factor antagonist infliximab for

For peer review only - http://bmjopen.bmj.com/site/about/guidelines.xhtml

treatment-resistant depression: the role of baseline inflammatory biomarkers. *JAMA psychiatry* 2013;70(1):31-41.

18. Capuron L, Fornwalt FB, Knight BT, Harvey PD, Ninan PT, Miller AH. Does cytokine-induced depression differ from idiopathic major depression in medically healthy individuals? *Journal of affective disorders* 2009;119(1-3):181-5.
19. Capuron L, Gumnick JF, Musselman DL, Lawson DH, Reemsnyder A, Nemeroff CB, et al. Neurobehavioral effects of interferon-alpha in cancer patients: phenomenology and paroxetine responsiveness of symptom dimensions. *Neuropsychopharmacology : official publication of the American College of Neuropsychopharmacology* 2002;26(5):643-52.
20. Jokela M, Virtanen M, Batty GD, Kivimaki M. Inflammation and Specific Symptoms of Depression. *JAMA psychiatry* 2016;73(1):87-8.
21. Ferrie JE, Kivimaki M, Akbaraly TN, Singh-Manoux A, Miller MA, Gimeno D, et al. Associations between change in sleep duration and inflammation: findings on C-reactive protein and interleukin 6 in the Whitehall II Study. *American journal of epidemiology* 2013;178(6):956-61.
22. Harrison NA, Voon V, Cercignani M, Cooper EA, Pessiglione M, Critchley HD. A Neurocomputational Account of How Inflammation Enhances Sensitivity to Punishments Versus Rewards. *Biological psychiatry* 2016;80(1):73-81.
23. Chu AL, Stochl J, Lewis G, Zammit S, Jones PB, Khandaker GM. Longitudinal Association between Inflammatory Markers and Somatic and Psychological Symptoms of Depression in a Prospective Birth Cohort. *(under review)* 2018.
24. Duivis HE, Vogelzangs N, Kupper N, de Jonge P, Penninx BW. Differential association of somatic and cognitive symptoms of depression and anxiety with inflammation: findings from the Netherlands Study of Depression and Anxiety (NESDA). *Psychoneuroendocrinology* 2013;38(9):1573-85.

For peer review only

For peer review only - http://bmjopen.bmj.com/site/about/guidelines.xhtml

25. Kaser M, Zaman R, Sahakian BJ. Cognition as a treatment target in depression. *Psychological medicine* 2017;47(6):987-89.

26. Sparkman NL, Buchanan JB, Heyen JR, Chen J, Beverly JL, Johnson RW. Interleukin-6 facilitates lipopolysaccharide-induced disruption in working memory and expression of other proinflammatory cytokines in hippocampal neuronal cell layers. *The Journal of neuroscience : the official journal of the Society for Neuroscience* 2006;26(42):10709-16.

27. Donegan JJ, Girotti M, Weinberg MS, Morilak DA. A novel role for brain interleukin-6: facilitation of cognitive flexibility in rat orbitofrontal cortex. *The Journal of neuroscience : the official journal of the Society for Neuroscience* 2014;34(3):953-62.

28. Tang MM, Lin WJ, Pan YQ, Guan XT, Li YC. Hippocampal neurogenesis dysfunction linked to depressive-like behaviors in a neuroinflammation induced model of depression. *Physiology & behavior* 2016;161:166-73.

29. Mackinnon N, Zammit S, Lewis G, Jones PB, Khandaker GM. Association between childhood infection, serum inflammatory markers and intelligence: findings from a population-based prospective birth cohort study. *Epidemiology and infection* 2018;146(2):256-64.

30. Krogh J, Benros ME, Jorgensen MB, Vesterager L, Elfving B, Nordentoft M. The association between depressive symptoms, cognitive function, and inflammation in major depression. *Brain, behavior, and immunity* 2014;35:70-6.

31. Goldsmith DR, Haroon E, Woolwine BJ, Jung MY, Wommack EC, Harvey PD, et al. Inflammatory markers are associated with decreased psychomotor speed in patients with major depressive disorder. *Brain, behavior, and immunity* 2016;56:281-8.

32. Chang HH, Lee IH, Gean PW, Lee SY, Chi MH, Yang YK, et al. Treatment response and cognitive impairment in major depression: association with C-reactive protein. *Brain, behavior, and immunity* 2012;26(1):90-5.

For peer review only - http://bmjopen.bmj.com/site/about/guidelines.xhtml

33. Chan AW, Tetzlaff JM, Altman DG, Laupacis A, Gotzsche PC, Krle AJK, et al. SPIRIT 2013 Statement: defining standard protocol items for clinical trials. *Rev Panam Salud Publica* 2015;38(6):506-14.

34. Calabrese LH, Rose-John S. IL-6 biology: implications for clinical targeting in rheumatic disease. *Nature reviews. Rheumatology* 2014;10(12):720-7.

35. Bonaccorso S, Marino V, Biondi M, Grimaldi F, Ippoliti F, Maes M. Depression induced by treatment with interferon-alpha in patients affected by hepatitis C virus. *Journal of affective disorders* 2002;72(3):237-41.

36. O'Connor JC, Lawson MA, Andre C, Moreau M, Lestage J, Castanon N, et al. Lipopolysaccharide-induced depressive-like behavior is mediated by indoleamine 2,3-dioxygenase activation in mice. *Molecular psychiatry* 2009;14(5):511-22.

37. Haroon E, Raison CL, Miller AH. Psychoneuroimmunology meets neuropsychopharmacology: translational implications of the impact of inflammation on behavior. *Neuropsychopharmacology : official publication of the American College of Neuropsychopharmacology* 2012;37(1):137-62.

38. Khandaker GM, Dantzer R. Is there a role for immune-to-brain communication in schizophrenia? *Psychopharmacology* 2016;233(9):1559-73.

39. Hodes GE, Pfau ML, Leboeuf M, Golden SA, Christoffel DJ, Bregman D, et al. Individual differences in the peripheral immune system promote resilience versus susceptibility to social stress. *Proceedings of the National Academy of Sciences of the United States of America* 2014;111(45):16136-41.

40. WHO. The ICD-10 Classification of Mental and Behavioural Disorder: Clinical Descriptions and Disgnostic Guidelines: World Health Organization, 1992.

41. Beck AT, Steer RA, Brown GK. Beck depression inventory-II. *San Antonio* 1996;78(2):490-8.

For peer review only

For peer review only - http://bmjopen.bmj.com/site/about/guidelines.xhtml

42. Wiles N, Thomas L, Abel A, Barnes M, Carroll F, Ridgway N, et al. Clinical effectiveness and cost-effectiveness of cognitive behavioural therapy as an adjunct to pharmacotherapy for treatment-resistant depression in primary care: the CoBalT randomised controlled trial. *Health technology assessment* 2014;18(31):1-167, vii-viii.

43. Kroenke K, Spitzer RL, Williams JB. The PHQ-9: validity of a brief depression severity measure. *J Gen Intern Med* 2001;16(9):606-13.

44. Woloshin S, Schwartz LM. Distribution of C-reactive protein values in the United States. *The New England journal of medicine* 2005;352(15):1611-3.

45. Woo P, Wilkinson N, Prieur AM, Southwood T, Leone V, Livermore P, et al. Open label phase II trial of single, ascending doses of MRA in Caucasian children with severe systemic juvenile idiopathic arthritis: proof of principle of the efficacy of IL-6 receptor blockade in this type of arthritis and demonstration of prolonged clinical improvement. *Arthritis research & therapy* 2005;7(6):R1281-8.

For peer review only

For peer review only - http://bmjopen.bmj.com/site/about/guidelines.xhtml

SPIRIT
STANDARD PROTOCOL ITEMS: RECOMMENDATIONS FOR INTERVENTIONAL TRIALS

SPIRIT 2013 Checklist: Recommended items to address in a clinical trial protocol and related documents*

Section/item	Item No	Description	Addressed on page number
**Administrative information**			
Title	1	Descriptive title identifying the study design, population, interventions, and, if applicable, trial acronym	1
Trial registration	2a	Trial identifier and registry name. If not yet registered, name of intended registry	3
	2b	All items from the World Health Organization Trial Registration Data Set	3 (ISRCTN1694254)
Protocol version	3	Date and version identifier	-
Funding	4	Sources and types of financial, material, and other support	23
Roles and responsibilities	5a	Names, affiliations, and roles of protocol contributors	1, 23
	5b	Name and contact information for the trial sponsor	23
	5c	Role of study sponsor and funders, if any, in study design; collection, management, analysis, and interpretation of data; writing of the report; and the decision to submit the report for publication, including whether they will have ultimate authority over any of these activities	24
	5d	Composition, roles, and responsibilities of the coordinating centre, steering committee, endpoint adjudication committee, data management team, and other individuals or groups overseeing the trial, if applicable (see Item 21a for data monitoring committee)	21

For peer review only

For peer review only - http://bmjopen.bmj.com/site/about/guidelines.xhtml

**Introduction**			
Background and rationale	6a	Description of research question and justification for undertaking the trial, including summary of relevant studies (published and unpublished) examining benefits and harms for each intervention	5-8
	6b	Explanation for choice of comparators	7-8
Objectives	7	Specific objectives or hypotheses	9-10
Trial design	8	Description of trial design including type of trial (eg, parallel group, crossover, factorial, single group), allocation ratio, and framework (eg, superiority, equivalence, noninferiority, exploratory)	10
**Methods: Participants, interventions, and outcomes**			
Study setting	9	Description of study settings (eg, community clinic, academic hospital) and list of countries where data will be collected. Reference to where list of study sites can be obtained	16
Eligibility criteria	10	Inclusion and exclusion criteria for participants. If applicable, eligibility criteria for study centres and individuals who will perform the interventions (eg, surgeons, psychotherapists)	13, Fig. 2
Interventions	11a	Interventions for each group with sufficient detail to allow replication, including how and when they will be administered	15-16
	11b	Criteria for discontinuing or modifying allocated interventions for a given trial participant (eg, drug dose change in response to harms, participant request, or improving/worsening disease)	18, 20
	11c	Strategies to improve adherence to intervention protocols, and any procedures for monitoring adherence (eg, drug tablet return, laboratory tests)	-
	11d	Relevant concomitant care and interventions that are permitted or prohibited during the trial	-
Outcomes	12	Primary, secondary, and other outcomes, including the specific measurement variable (eg, systolic blood pressure), analysis metric (eg, change from baseline, final value, time to event), method of aggregation (eg, median, proportion), and time point for each outcome. Explanation of the clinical relevance of chosen efficacy and harm outcomes is strongly recommended	7-8, 16, Table 1
Participant timeline	13	Time schedule of enrolment, interventions (including any run-ins and washouts), assessments, and visits for participants. A schematic diagram is highly recommended (see Figure)	Fig. 2, 15-16

For peer review only - http://bmjopen.bmj.com/site/about/guidelines.xhtml

Sample size	14	Estimated number of participants needed to achieve study objectives and how it was determined, including clinical and statistical assumptions supporting any sample size calculations	14
Recruitment	15	Strategies for achieving adequate participant enrolment to reach target sample size	15
**Methods: Assignment of interventions (for controlled trials)**			
Allocation:			
Sequence generation	16a	Method of generating the allocation sequence (eg, computer-generated random numbers), and list of any factors for stratification. To reduce predictability of a random sequence, details of any planned restriction (eg, blocking) should be provided in a separate document that is unavailable to those who enrol participants or assign interventions	14
Allocation concealment mechanism	16b	Mechanism of implementing the allocation sequence (eg, central telephone; sequentially numbered, opaque, sealed envelopes), describing any steps to conceal the sequence until interventions are assigned	14
Implementation	16c	Who will generate the allocation sequence, who will enrol participants, and who will assign participants to interventions	14
Blinding (masking)	17a	Who will be blinded after assignment to interventions (eg, trial participants, care providers, outcome assessors, data analysts), and how	14
	17b	If blinded, circumstances under which unblinding is permissible, and procedure for revealing a participant's allocated intervention during the trial	20
**Methods: Data collection, management, and analysis**			
Data collection methods	18a	Plans for assessment and collection of outcome, baseline, and other trial data, including any related processes to promote data quality (eg, duplicate measurements, training of assessors) and a description of study instruments (eg, questionnaires, laboratory tests) along with their reliability and validity, if known. Reference to where data collection forms can be found, if not in the protocol	16, Table 1
	18b	Plans to promote participant retention and complete follow-up, including list of any outcome data to be collected for participants who discontinue or deviate from intervention protocols	-

For peer review only - http://bmjopen.bmj.com/site/about/guidelines.xhtml

Data management	19	Plans for data entry, coding, security, and storage, including any related processes to promote data quality (eg, double data entry; range checks for data values). Reference to where details of data management procedures can be found, if not in the protocol	21-22
Statistical methods	20a	Statistical methods for analysing primary and secondary outcomes. Reference to where other details of the statistical analysis plan can be found, if not in the protocol	14
	20b	Methods for any additional analyses (eg, subgroup and adjusted analyses)	14
	20c	Definition of analysis population relating to protocol non-adherence (eg, as randomised analysis), and any statistical methods to handle missing data (eg, multiple imputation)	-
**Methods: Monitoring**			
Data monitoring	21a	Composition of data monitoring committee (DMC); summary of its role and reporting structure; statement of whether it is independent from the sponsor and competing interests; and reference to where further details about its charter can be found, if not in the protocol. Alternatively, an explanation of why a DMC is not needed	21
	21b	Description of any interim analyses and stopping guidelines, including who will have access to these interim results and make the final decision to terminate the trial	N/A
Harms	22	Plans for collecting, assessing, reporting, and managing solicited and spontaneously reported adverse events and other unintended effects of trial interventions or trial conduct	20
Auditing	23	Frequency and procedures for auditing trial conduct, if any, and whether the process will be independent from investigators and the sponsor	-
**Ethics and dissemination**			
Research ethics approval	24	Plans for seeking research ethics committee/institutional review board (REC/IRB) approval	20
Protocol amendments	25	Plans for communicating important protocol modifications (eg, changes to eligibility criteria, outcomes, analyses) to relevant parties (eg, investigators, REC/IRBs, trial participants, trial registries, journals, regulators)	-

For peer review only - http://bmjopen.bmj.com/site/about/guidelines.xhtml

Consent or assent	26a	Who will obtain informed consent or assent from potential trial participants or authorised surrogates, and how (see Item 32)	21
	26b	Additional consent provisions for collection and use of participant data and biological specimens in ancillary studies, if applicable	21
Confidentiality	27	How personal information about potential and enrolled participants will be collected, shared, and maintained in order to protect confidentiality before, during, and after the trial	21-22
Declaration of interests	28	Financial and other competing interests for principal investigators for the overall trial and each study site	24
Access to data	29	Statement of who will have access to the final trial dataset, and disclosure of contractual agreements that limit such access for investigators	-
Ancillary and post-trial care	30	Provisions, if any, for ancillary and post-trial care, and for compensation to those who suffer harm from trial participation	-
Dissemination policy	31a	Plans for investigators and sponsor to communicate trial results to participants, healthcare professionals, the public, and other relevant groups (eg, via publication, reporting in results databases, or other data sharing arrangements), including any publication restrictions	22
	31b	Authorship eligibility guidelines and any intended use of professional writers	-
	31c	Plans, if any, for granting public access to the full protocol, participant-level dataset, and statistical code	-
**Appendices**			
Informed consent materials	32	Model consent form and other related documentation given to participants and authorised surrogates	Appendix 1
Biological specimens	33	Plans for collection, laboratory evaluation, and storage of biological specimens for genetic or molecular analysis in the current trial and for future use in ancillary studies, if applicable	-

*It is strongly recommended that this checklist be read in conjunction with the SPIRIT 2013 Explanation & Elaboration for important clarification on the items. Amendments to the protocol should be tracked and dated. The SPIRIT checklist is copyrighted by the SPIRIT Group under the Creative Commons "Attribution-NonCommercial-NoDerivs 3.0 Unported" license.

For peer review only - http://bmjopen.bmj.com/site/about/guidelines.xhtml

BMJ Open

Protocol for the Insight Study: a randomised controlled trial of single dose tocilizumab in patients with depression and low-grade inflammation

Journal:	*BMJ Open*
Manuscript ID	bmjopen-2018-025333.R1
Article Type:	Protocol
Date Submitted by the Author:	14-Aug-2018
Complete List of Authors:	Khandaker, Golam; University of Cambridge School of Clinical Medicine, Psychiatry<br>Oltean, Bianca; University of Cambridge School of Clinical Medicine, Psychiatry<br>Kaser, Muzaffer; University of Cambridge School of Clinical Medicine, Psychiatry<br>Dibben, Claire; Norfolk and Suffolk NHS Foundation Trust<br>Ramana, Rajini; Cambridgeshire and Peterborough NHS Foundation Trust<br>Jadon, Deepak; Cambridge University Hospitals NHS Foundation Trust<br>Dantzer , R ; Department of Symptom Research, MD Anderson Cancer Center, Houston<br>Coles, Alasdair; Department of Clinical Neurosciences, University of Cambridge<br>Lewis, Glyn; UCL Psychiatric Epidemiology, Mental Health Sciences Unit<br>Jones, Peter; University of Cambridge, Department of Psychiatry
**Primary Subject Heading**:	Mental health
Secondary Subject Heading:	Mental health
Keywords:	Depression & mood disorders < PSYCHIATRY, Clinical trials < THERAPEUTICS, Protocols & guidelines < HEALTH SERVICES ADMINISTRATION & MANAGEMENT

SCHOLARONE™
Manuscripts

Protocol for the Insight Study: a randomised controlled trial of single dose tocilizumab in patients with depression and low-grade inflammation

Golam M Khandaker[1,2], Bianca P Oltean[1,2], Muzaffer Kaser[1,2], Claire R M Dibben[3], Rajini Ramana[1,2], Deepak R Jadon[4], Robert Dantzer[5], Alasdair Coles[6], Glyn Lewis[7], Peter B Jones[1,2]

[1] Department of Psychiatry, University of Cambridge, Cambridge, UK

[2] Cambridgeshire and Peterborough NHS Foundation Trust, Cambridge, UK

[3] Norfolk and Suffolk NHS Foundation Trust, Bury St Edmunds, UK

[4] Department of Rheumatology, Cambridge University Hospitals NHS Foundation Trust, Cambridge, UK

[5] Department of Symptom Research, MD Anderson Cancer Centre, University of Texas, Huston, USA

[6] Department of Clinical Neurosciences, University of Cambridge, Cambridge, UK

[7] Division of Psychiatry, University College London, London, UK

Correspondence to: Dr Golam Khandaker; Email: gmk24@medschl.cam.ac.uk

Word count:

Abstract: 259

Manuscript: 3497

Tables and Figures: 2 Tables and 2 Figures (plus one online supplementary Table)

For peer review only - http://bmjopen.bmj.com/site/about/guidelines.xhtml

ABSTRACT

Introduction

Observational studies indicate a potentially causal role for interleukin 6 (IL-6), a proinflammatory cytokine, in pathogenesis of depression, but interventional studies based on patients with depression have not been conducted. Tocilizumab, anti-inflammatory drug, is a humanised monoclonal antibody that inhibits IL-6 signalling and is licensed in the UK for treatment of rheumatoid arthritis. The main objectives of this study are to test whether IL-6 contributes to the pathogenesis of depression, and to examine potential mechanisms by which IL-6 affects mood and cognition. A secondary objective is to compare depressed participants with and without evidence of low-grade systemic inflammation.

Methods and analysis

This is a proof-of-concept, randomised, parallel group, double blind, placebo-controlled clinical trial. Approximately 50 participants with ICD-10 diagnosis of depression who have evidence of low-grade inflammation, defined as serum high sensitivity C-reactive protein (hsCRP) level ≥3mg/L, will receive either a single intravenous infusion of tocilizumab or normal saline. Blood samples, behavioural and cognitive measures will be collected at baseline and after infusion around day 7, 14 and 28. The primary outcome is somatic symptoms score around day 14 post-infusion. In addition, approximately 50 depressed participants without low-grade inflammation (serum hsCRP level <3mg/L) will complete the same baseline assessments as the randomised cohort.

Ethics and dissemination

The study has been approved by the South Central - Oxford B Research Ethics Committee (Reference: 18/SC/0118). Study findings will be published in peer-review journals. Findings will be also disseminated by conference/departmental presentations, and by social and traditional media.

For peer review only - http://bmjopen.bmj.com/site/about/guidelines.xhtml

Trial registration number

ISRCTN16942542 (prospective registration pre first participant first visit).

Link: http://www.isrctn.com/ISRCTN16942542

Key words

Depression; Somatic Symptoms; Interleukin 6; Interleukin 6 Receptor; Tocilizumab; Monoclonal Antibody; Randomised Controlled Trial; Clinical Trial; Immunopsychiatry

For peer review only

For peer review only - http://bmjopen.bmj.com/site/about/guidelines.xhtml

Strengths and limitations of this study

- This is one of the first studies to examine the role of IL-6 in patients with depression using a randomised controlled trial design.
- The study examines important intermediate markers for antidepressant effect including somatic symptoms of depression, which will provide mechanistic insights into potential role of inflammation in depression.
- Comparison between inflamed and non-inflamed patients will help to better characterise patients with inflammation-related depression.
- As single dose of tocilizumab will be used, the study may not be able to test conclusively the efficacy of tocilizumab as potential treatment for depression.

For peer review only

For peer review only - http://bmjopen.bmj.com/site/about/guidelines.xhtml

INTRODUCTION

Scientific Background and Study Rationale

Low-grade systemic inflammation as reflected by elevated concentrations of circulating inflammatory markers in peripheral blood may play a role in pathogenesis of depression[1 2]. Meta-analysis of cross-sectional studies confirm that concentrations of circulating inflammatory cytokines, such as interleukin 6 (IL-6) and acute phase proteins, such as C-reactive protein (CRP), are elevated in acutely unwell patients with depression[3-5], which tend to normalise after recovery[5] but continue to be elevated in treatment resistant patients[6 7]. Experimental studies indicate IL-6 and other cytokines are important mediators of the effects of inflammation on the brain[1 2]. However, it is unclear whether inflammation plays a causal role in depression because cytokine elevation could be a consequence of depression (i.e. reverse causality) or due to confounding. Existing epidemiological studies have addressed these issues to some extent (below), but randomised controlled trials (RCT) of anti-cytokine treatment in depression are scarce. Intervention studies targeting the IL-6 system are required for a better understanding of the relationship between inflammation and depression.

Population-based longitudinal studies showing an association between elevated concentrations of IL-6 or CRP at baseline and increased risk of depression at follow-up indicates reverse causality is an unlikely explanation for previously observed association between IL-6 and depression [8-11]. Using data from the Avon Longitudinal Study of Parents and Children (ALSPAC) birth cohort, we have reported that elevated concentrations of serum IL-6 in childhood are associated with increased risk of depression subsequently in early-adulthood in a linear, dose-response fashion[9] (Figure 1, Panel A). Evidence for this association remains after controlling for potential confounders including sex, body mass index, and maternal depression. This is one of the first evidence from humans that low-grade inflammation precedes depression, so is unlikely to be simply a consequence of illness.

For peer review only - http://bmjopen.bmj.com/site/about/guidelines.xhtml

Results from genetic association analysis informed by Mendelian randomization (MR) indicate residual confounding is unlikely to explain the association between IL-6 and depression fully. MR is based on the idea that if a biomarker is causally related to an illness, genetic variant(s) regulating levels/activity of that biomarker should also be associated with the illness[12 13]. Genetic variants segregate at random during meiosis and are unrelated to potential confounders, so using them as markers of exposure could overcome confounding. Using data from the ALSPAC birth cohort, we have shown that a genetic variant in the IL-6 receptor gene (*IL6R* Asp358Ala; rs2228145) that is known to dampen down inflammation by impairing the activity of IL-6 is protective for severe depression[14] (Figure 1, Panel B). The genetic variant is strongly associated with serum IL-6 and CRP levels, but not with any common confounders of the inflammation-depression relationship such as sex, social class, ethnicity, and body mass index. These findings strongly indicate that the IL-6/IL-6R pathways play a role in the pathophysiology of depression.

Although human population-based observational studies strongly support an association between IL-6 and depression, observational studies cannot confirm causality. RCTs are needed to test whether manipulation of IL-6 signalling has an impact on depressive symptoms in individuals with depression, but such studies are lacking. RCTs could also elucidate potential mechanisms by which IL-6 affects mood and cognition. Inflammation is unlikely to be relevant for all patients with depression[15], so consideration is required regarding the choice of suitable patients and outcomes for clinical trials of anti-inflammatory treatment to elucidate potential mechanistic role of the IL-6 system in depression (below).

Stratified Patient Selection and Choice of Outcomes

A meta-analysis has reported that anti-cytokine drugs improve depressive symptoms in patients with chronic inflammatory illness, such as rheumatoid arthritis, independently of

For peer review only - http://bmjopen.bmj.com/site/about/guidelines.xhtml

improvement in physical illness[16]. Similarly, an RCT of infliximab (anti-TNF monoclonal antibody and anti-inflammatory drug), which excluded patients with chronic physical illness, reported that the drug is more likely to improve depressive symptoms in depressed patients who show evidence of low-grade inflammation (i.e., elevated CRP levels) at baseline[17]. Therefore, clinical trials of IL-6 modulation should focus on depressed participants who have evidence of low-grade inflammation. Patients who do not get better with antidepressants are more likely to show evidence of low-grade inflammation[6 7].

Inflammatory cytokines are more likely to be relevant for somatic symptoms of depression (e.g. fatigue, appetite and sleep disturbance) rather than psychological symptoms (e.g. hopelessness). Fatigue, sleep disturbance develop rapidly in majority of interferon-treated cancer patients who develop depression (an established human model for inflammation-induced depression), but cognitive and affective symptoms (e.g. impaired memory, low mood) develop slowly and relatively less frequently[18 19]. Population-based studies have shown that elevated serum IL-6 and CRP levels are associated with fatigue, impaired sleep, but not with hopelessness[20 21]. Cytokine-induced somatic symptoms may affect mood by reducing rewarding experiences[22], so could be a mediator of the relationship between inflammation and depression. This idea is consistent with our own work from the ALSPAC birth cohort which indicates that somatic symptoms mediate the association between IL-6 and psychological symptoms[23]. Other groups have also reported that somatic symptoms of depression are associated with CRP, IL-6, and TNF alpha levels[24]. Therefore, somatic symptoms could be useful treatment target and marker of treatment response in clinical trials of anti-inflammatory treatment for depression. However, to our knowledge no interventional study has examined the effects of reducing IL-6 activity on somatic symptoms specifically in individuals with depression.

For peer review only - http://bmjopen.bmj.com/site/about/guidelines.xhtml

Cognitive dysfunction, an unmet treatment need in depression[25], could be related to inflammation. Preclinical studies suggest that IL-6 may mediate inflammation-induced cognitive dysfunction in rats and mice[26 27]. Neuroinflammation is associated with depressive symptoms and increased production of inflammatory cytokines in the hippocampus, a bran structure critical for memory[28]. At population level, associations between circulating IL-6, CRP and general intelligence[29] and cognitive symptoms of depression[8] has been reported. In patients with depression, higher inflammatory marker levels are associated with poor psychomotor speed[30 31] and persistent cognitive dysfunction[32]. Therefore, in addition to depressive symptoms, inclusion of measures of cognitive function could provide useful insights into potential role of inflammation in depression.

Proposed Study

We propose a proof-of-concept, randomised, parallel group, double blind, placebo controlled clinical trial to investigate whether IL-6 contributes to pathogenesis of depression, and to examine potential mechanisms by which IL-6 affects mood and cognition. We propose that inhibition of IL-6 signalling in individuals with depression who show evidence of low-grade inflammation and poor response to antidepressants would attenuate their depressive symptoms particularly somatic symptoms of depression.

Patients with depression who show evidence of inflammation may be different from those who do not. Those with low-grade inflammation are likely to be antidepressant resistant[6 7], so a clearer understanding of this group would be clinically useful. We also propose an observational study to examine similarities and differences between depressed patients with and without low-grade inflammation.

For peer review only - http://bmjopen.bmj.com/site/about/guidelines.xhtml

STUDY AIMS AND HYPOTHESIS

Primary

To carry out a proof-of-concept, randomized, parallel group, double blind, placebo-controlled clinical trial to test whether IL-6 contributes to pathogenesis of depression, and to examine potential mechanisms by which IL-6 affects mood and cognition. We hypothesise that inhibition of IL-6 signalling with a single intravenous (IV) infusion of anti-IL6R monoclonal antibody tocilizumab will attenuate somatic symptoms of depression, improve cognitive function, reduce serum proinflammatory cytokine levels and IDO activation in individuals with depression who show evidence of low-grade inflammation and poor response to antidepressant. Low-grade inflammation, hereafter also referred to as 'inflamed depression', will be defined as serum high sensitivity CRP (hsCRP) level ≥3mg/L.

Secondary

To carry out an observational study to examine differences and similarities between inflamed and non-inflamed depression (CRP <3mg/L). We hypothesise that individuals with inflamed depression, compared with non-inflamed, will be more likely to have somatic symptoms, higher levels of serum proinflammatory cytokines, cognitive dysfunction and evidence of IDO activation.

METHODS

This protocol has been prepared in accordance with the Standard Protocol Items: Recommendations for Interventional Trials (SPIRIT) 2013 statement[33]. Please see eTable 1 for the SPIRIT checklist.

For peer review only - http://bmjopen.bmj.com/site/about/guidelines.xhtml

Patient and public involvement

Patients were consulted during the development of the study protocol who contributed to the procedure, consent form, and information leaflets. The study information was made more accessible and data collection assessments shorter. Interested participants will receive a summary of the main findings at the end of the study.

Study design and Sample

The study has two parts. The clinical trial part will include approximately 50 depressed patients with CRP ≥3mg/L (intervention cohort) who will be randomised into two groups (tocilizumab or normal saline). The observational part will compare the intervention cohort at baseline with 50 non-inflamed depressed patients (CRP <3mg/L). Non-inflamed patients will not be randomised. For an overview of the study design please see Figure 2.

Intervention

The study intervention will be a single IV infusion of tocilizumab (8mgs/kg; max 800mgs/patient) or normal saline. Tocilizumab is the first-in-class, anti-IL-6R humanized monoclonal antibody, which is commercially available and licensed in the UK for treating rheumatoid arthritis and juvenile idiopathic arthritis. Tocilizumab blocks both IL-6 classic and trans-signaling (responsible for most of the inflammatory effects of IL-6) making it the agent of choice for complete IL-6 inhibition[34]. As justified by interferon[35] and mouse[36] studies, peripheral immune-activation causes depression because IL-6 and other circulating cytokines can influence the brain using neural, humoral and cellular pathways[1 2 37 38]. Therefore, tocilizumab which is mostly peripherally acting is likely to have an impact on symptoms of depression. Infliximab, an anti-TNF-α monoclonal antibody, that has similar, limited blood-brain barrier penetration as tocilizumab has been reported to modulate

For peer review only - http://bmjopen.bmj.com/site/about/guidelines.xhtml

symptoms of depression[17]. Similarly, peripherally acting anti-IL-6 monoclonal antibody has been reported to reduce depression-like behaviour in mice exposed to repeated stress[39].

Eligibility criteria

The study will include adult participants aged 20 to 65 years meeting ICD-10 criteria[40] for depressive episode who are currently taking an antidepressant at adequate dose (according to BNF) for at least four weeks. In addition, those included in the clinical trial part will have serum hsCRP levels ≥3mg/L. Please see Table 1 for complete inclusion and exclusion criteria.

Table 1: Insight Study Inclusion and Exclusion Criteria

Group	Inclusion criteria	Exclusion criteria
**All participants**	• Provide informed consent<br>• Understand written and spoken English<br>• Able to consent to blood sampling<br>• Willing to abstain from strenuous exercise for 72 hours prior to assessment<br>• **Age:** 20-65 years (inclusive)<br>• **Diagnosis of depression:** meet ICD-10 criteria at the time of assessment<br>• **Somatic symptom score:** ≥7 at the time of eligibility based on Beck Depression Inventory II (BDI II) items 4=lack of pleasure, 15=loss of energy, 16=changes in sleeping pattern, 18=changed in appetite, 19=concentration difficulty, 20=tiredness or fatigue, and 21=loss of interest in sex	• Current or lifetime diagnosis of bipolar disorder, psychotic disorder, personality disorder or eating disorder<br>• Current suicidal thoughts or history of suicide attempt, deliberate self-harm, overdose within six months prior to eligibility assessment<br>• History of alcohol or substance use disorder (abuse/dependence) within six months prior to eligibility assessment<br>• Pregnant or breast feeding<br>• History of serious allergic reaction after any infusion<br>• Current use of medication likely to compromise interpretation of immunological data (including, but not limited to, antibiotics, non-steroidal anti-inflammatory drugs, oral/injectable corticosteroids)<br>• Any major episode of infection requiring hospitalization or treatment with IV antibiotics within 4 weeks of eligibility assessment<br>• Presence or history of the following illnesses: recurrent bacterial, viral, fungal, mycobacterial or other opportunistic infections; unstable cardiac, pulmonary, renal, hepatic, endocrine, hematologic, or active infectious disease; rheumatic autoimmune

For peer review only - http://bmjopen.bmj.com/site/about/guidelines.xhtml

	• **History of non/slow response to antidepressant:** receiving treatment with an antidepressant at adequate dose (according to BNF) for at least four weeks	disease, mixed connective tissue disease, scleroderma, polymyositis; uncontrolled hypertension<br>• No history of chicken pox infection or no history of varicella zoster vaccination
**Intervention Cohort**	• Serum/plasma hsCRP level ≥3mg/L	• Current or past infection with TB, Hepatitis B, Hepatitis C, HIV or VZV<br>• History of severe allergic or anaphylactic reactions to human, humanized or murine monocloncal antibodies

For peer review only

Outcome

The primary outcome is change in total somatic symptom score from baseline to around day 14 post-infusion assessed using the Beck Depression Inventor-II[41] (BDI-II). The somatic symptom score will be constructed by summing scores for seven relevant BDI-II items (4=lack of pleasure, 15=loss of energy, 16=changes in sleeping pattern, 18=changes in appetite, 19=concentration difficulty, 20=tiredness or fatigue, and 21=loss of interest in sex). Depression severity is secondary outcome also assessed by BDI II. As an experimental medicine study, we will collect data on a range of tertiary/exploratory measures including, but not limited to, fatigue, anhedonia, cognitive function, and peripheral blood analyses of inflammatory markers, cortisol and markers of IDO activation.

Statistical power

No RCTs were available to inform a power calculation for the proposed primary outcome. However, the clinical trial (N=50) will have 80% statistical power (alpha=0.05) to detect a 2.5-point reduction in clinical interview schedule revised (CIS-R) depression score in tocilizumab group compared with placebo; mean (SD) for outcome=15(3) based on a previous RCT of depression[42]. We believe the actual sample size needed for the primary outcome will be smaller, as somatic symptoms are strongly influenced by inflammation.

For peer review only - http://bmjopen.bmj.com/site/about/guidelines.xhtml

Randomisation and blinding

Participants will be randomly assigned to tocilizumab or normal saline group (1:1) using minimization method to ensure that groups are comparable on somatic symptom severity and sex. Sealed Envelope, an external company, will do randomization. Randomization codes will be sent directly to the Central Pharmacy, Addenbrooke's Hospital, Cambridge University Hospitals NHS Foundation Trust, who will dispense tocilizumab or normal saline according to randomization schedule. Infusions will be prepared and administered at a clinical research facility (CRF) in Cambridge Biomedical Campus by CRF staff. Infusion packs containing drug or placebo will be visually indistinguishable ensuring both participants and study team remain blind about allocation of intervention.

Statistical analysis

The clinical trial data will be analysed using an intention-to-treat approach. We will compare outcome measures between treatment and placebo groups controlling for baseline scores. This mechanistic experiment will focus on overall pattern of results rather than *P*-values for individual tests of statistical significance. Analysis for the secondary observational study will compare psychiatric, cognitive, and biomarkers between inflamed and non-inflamed groups using appropriate parametric and non-parametric statistical tests.

For peer review only

STUDY PROCEDURE

Identification of potentially eligible participants

An overview of study procedures has been presented in Figure 2. The study has been adopted by the National Institute for Health Research (NIHR) clinical research network (CRN) portfolio. Recruitment sites will include Cambridgeshire and Peterborough NHS Foundation Trust (CPFT), Norfolk and Suffolk NHS Foundation Trust (NSFT), and primary care general practices (GP) in Cambridgeshire and Suffolk. In addition, we will advertise in

For peer review only - http://bmjopen.bmj.com/site/about/guidelines.xhtml

social media and in the community. Potentially eligible participants will be identified directly by clinicians, and by electronic search of clinical/research databases used by CPFT and GP surgeries. Participants will be first approached by their clinicians unless they had previously given consent to be contacted for research.

Eligibility assessment

Face-to-face assessment will be carried out to establish eligibility and to obtain informed consent. A blood sample will be collected for serum hsCRP measurement. In addition, participants identified from primary care will be first screened using a questionnaire containing key inclusion/exclusion criteria and the patient health questionnaire 9 (PHQ-9)[43].

Baseline data collection

All participants (50 inflamed and 50 non-inflamed) will attend a face-to-face assessment comprising psychiatric measures, cognitive tasks and blood sampling. Please see Table 2 for a list of all study measures. This will be the final study contact for non-inflamed participants. Inflamed participants will undergo further tests to establish safety/eligibility to receive tocilizumab, which will include a chest X-ray and blood tests to exclude pregnancy and certain infections, such as TB, HIV. Eligible participants will be randomised and will be invited for infusion.

For peer review only - http://bmjopen.bmj.com/site/about/guidelines.xhtml

Table 2: Study Measures

Domain	Tool	Source	Validated tool	Time of assessment
**Socio-demographic/ lifestyle**	Screening questionnaire	Self-report		Screening
	Sociodemographic questionnaire	Self-report		Baseline
	Antidepressant history & concomitant treatment questionnaire	Self-report/GP		Baseline
	Drug and alcohol questionnaire	Self-report		Baseline
**Psychiatric**	Patient Health Questionnaire 9	Self-report	✓	Screening
	Clinical Interview Schedule-Revised	Self-report	✓	Eligibility
	Beck Depression Inventory-II	Self-report	✓	Eligibility, Baseline, Follow-ups
	Snaith-Hamilton Pleasure Scale Questionnaire	Self-report	✓	Baseline, Follow-ups
	Multi-dimensional Fatigue Inventory	Self-report	✓	Baseline, Follow-ups
	Visual Analogue Scales for Subjective Feelings	Self-report		Baseline, Follow-ups
	Perceived Stress Scale	Self-report	✓	Baseline
**Cognitive**	National Adult Reading Scale for estimated premorbid IQ	Interviewer-assessed	✓	Baseline, Follow-up 2
	CANTAB Reaction Time	Computer task	✓	Baseline, Follow-up 2
	Symbol Coding Task	Computer / Paper		Baseline, Follow-up 2
	CANTAB Rapid Visual Information Processing	Computer task	✓	Baseline, Follow-up 2
	CANTAB Paired Associates Learning	Computer task	✓	Baseline, Follow-up 2
	Emotional categorization and recall task	Computer task	✓	Baseline, Follow-up 2
**Biologic**	Inflammatory markers, cardio-metabolic markers, IDO activation, White Cell phenotyping	Laboratory tests		Baseline, Follow-ups
**Genetic**	Gene expression/ Genotyping	Blood (RNA, DNA)		Baseline, Follow-up 2

Intervention

For peer review only - http://bmjopen.bmj.com/site/about/guidelines.xhtml

Intravenous infusion of tocilizumab or normal saline will be given continuously over an hour at a CRF in Cambridge Biomedical Campus by trained clinical staff under the supervision of a designated study doctor. Participants will remain under clinical observation for a further one-hour period after the end of infusion.

Follow-up

Follow-up assessments will take place approximately 7, 14 and 28 days after infusion, and will be similar to baseline data collection. Cognitive tasks will be administered only on day 14. At around 42 days after infusion, participants will be contacted by phone to provide final debrief at which point they will exit the study.

RISK MANAGEMENT

Depression-related Risks

To minimise risk, all participants are required to be registered with a general practitioner, and give consent to access GP records to verify eligibility and to share clinically relevant findings with their GP. If a participant becomes distressed during an interview, or does not wish to continue for any reason, the researcher will stop the interview and liaise with the study doctor to decide on appropriate course of action. Participants will be assessed for suicidality during eligibility assessment. Those with serious suicidal thoughts or history of suicide attempt, deliberate self-harm, overdose in six months prior to eligibility assessment will be excluded.

Procedure-related risks

For peer review only - http://bmjopen.bmj.com/site/about/guidelines.xhtml

Venepuncture

The study requires blood draw. Blood taking is associated with mild discomfort and bruising though serious side effects are rare. Efforts will be made to minimise risk. Blood taking will be performed by a nurse, doctor or research team member trained in venepuncture.

Chest X-ray

Participants in the intervention cohort will receive a chest x-ray to screen for Tuberculosis with a typical effective radiation dose of 0.016 mSv. This x-ray is additional to any standard clinical care outside of the trial. The dose is equivalent to that received, on average in the UK, from natural sources of radiation in the environment every three to ten days. All examinations will need to be completed in compliance with local Ionising Radiation Medical Exposure Regulations Employer's Procedures.

CRP Levels

Participants will be informed of their serum hsCRP level. The proposed threshold for defining participants as 'inflamed' used in this study is serum CRP level ≥3mg/L. Having serum hsCRP level above this threshold is not necessarily a cause for concern. About 30% of the general population have serum CRP levels 3.0-9.9mg/L, and about 10-15% have levels >10mg/L[44]. Reasons for elevated CRP in the absence of an infection or chronic inflammatory illness could include obesity, smoking, alcohol use, lack of exercise, so knowledge of CRP levels might prompt participants to adopt a healthier lifestyle. If serum CRP level is very high (>20mg/L) without any apparent explanation such as infection or chronic inflammatory illness, we will inform the GP and the participant will be excluded from the study.

Risks to research staff

For peer review only - http://bmjopen.bmj.com/site/about/guidelines.xhtml

When home visits or lone working are required, staff will follow local safety procedures. Home visits will be conducted in pairs whenever possible for first visits.

Safety considerations for infusion and monitoring of adverse reaction

Before infusion

Participants will be selected based on strict inclusion and exclusion criteria to minimise risk. In addition, we will carry out blood/other tests to exclude TB, HIV, VZV, Hepatitis B and C because, though unlikely after a single dose, tocilizumab infusion could make these infections worse. Female participants of childbearing age will be given blood test for pregnancy. Participants who are sexually active will be asked to use contraception for six weeks after infusion. Male participants will be also asked not to donate sperm samples for six weeks after infusion.

During infusion

Infusions will be given under supervision of a designated study doctor. Participants will be monitored throughout the duration of infusion; vital signs and possible side effects will be recorded. Any immediate adverse events will be managed in line with the Addenbrooke's Clinical Research Centre adverse event policy (ACRC/SOP018).

After infusion

Participants will remain under observation for a one-hour period after infusion. Participants will be advised to seek help through GP or A&E if they feel unwell after leaving the hospital. Before leaving the CRF, participants will be given an information sheet containing a telephone number their health professionals can call. If necessary we will un-blind the participant and inform their health professional whether the participant had received tocilizumab or normal saline. Adverse reactions will be recorded at every follow-up and at

For peer review only - http://bmjopen.bmj.com/site/about/guidelines.xhtml

final contact over the phone on day 42 after infusion. The expected time for complete elimination of tocilizumab from the system is approximately 42 days after a single infusion[45].

ETHICS AND DISSEMINATION

The study has been approved by the South Central - Oxford B Research Ethics Committee (REC) on 24/04/2018 (Reference: 18/SC/0118), and Health Research Authority (HRA) on 02/05/2018 (IRAS ID: 238297). The study will be conducted in accordance with guidelines from the REC, HRA and local R&D departments. The study team will prepare protocol amendments as required and ethics approval will be sought before implementing any changes to the approved protocol. The ISRCTN Trial Registry and the Research Governance Office will be informed of any amendments to the protocol.

Consent

Informed consent will be obtained for screening and for participation in the study. This will include consent to randomise, to contact GP to inform about participation in study, to access GP/psychiatric records to verify medical history to establish eligibility, and to inform GP any results/outcomes as necessary. Consent for additional tests to establish safety for tocilizumab infusion and for storing biological samples will be also obtained. Participants have the option to opt in to being contacted for future studies.

Study management

The CI will have overall responsibility for the study. A named principal investigator (PI) will take clinical responsibility for the research team at each site. The CI will meet the study team regularly to discuss any issues. The study does not require the formal arrangement of a steering committee, because, according to HRA, it is not a Clinical Trial of an Investigational Medicinal Product (CTIMP). However, to enhance monitoring of study, a Study Management

For peer review only - http://bmjopen.bmj.com/site/about/guidelines.xhtml

Group has been set up, which includes academic and clinical experts in psychiatry, rheumatology, neuroscience and immunology.

Data management and retention of samples

All potential participants will be assigned a unique study specific participant ID number. All data including personal details identifying individuals and anonymised data will be subject to good practice as laid down in the Data Protection Act. Each stage of inviting, informing and assessing a particular participant is tracked so that their (anonymised) current status within the study is known and assessment and other appointment dates are forecasted. This information is held on the secure, password protected database. Anonymised data from assessments will be uploaded to the secure, password protected database using web-based data entry systems, transcribing from paper copy as necessary. Minimal personal data (e.g., date of birth, sex) could be indexed by each participant's unique ID number.

Blood samples collected in this study may be stored for up to 10 years after the completion of the study for additional research. Stored samples will be coded throughout the sample storage and analysis process and will not be labelled with personal identifiers. Participants may withdraw their consent for their samples to be stored for future research.

For peer review only

Dissemination plan

Study findings will be published in peer-review journals. Publications will conform to the guidelines of the International Committee of Medical Journal Editors (ICMJE). Findings will be disseminated at conferences, departmental talks and other scientific meetings. Further dissemination will take place via social and traditional media.

For peer review only - http://bmjopen.bmj.com/site/about/guidelines.xhtml

Acknowledgements

The authors would like to thank NIHR CRN Eastern mental health and primary care teams for their help with preparing study protocol and materials. We are grateful to Ruth Holmes and Clare Knight from CRN for organising reviews by patient representatives of a previous version of the study protocol, and the anonymous patient advisors for their useful comments and suggestions. We are grateful to Jane Gaffa from CPFT R&D dept. for her help with study approvals, and to Dr Joel Parkinson, Department of Psychiatry, University of Cambridge, for his help with revising this manuscript.

Contributors

GMK designed the study, obtained funding, developed protocol and study materials, obtained necessary approvals for the study, and revised this manuscript. BPO contributed to study design, developed protocol and study materials, obtained necessary approvals for the study, and wrote first draft for this manuscript. PBJ contributed to study design, obtaining funding, development of the protocol and revised this manuscript. AC, DRJ, GL, MK, CRMD, RD and RR contributed to study design, protocol and revised this manuscript. Coles, Dantzer, Jadon, Jones, Khandaker, Lewis, and Ramana are part of the Study Management Group. GMK is the chief investigator, lead researcher and guarantor of the study.

Funding

The study is funded by Wellcome Trust Intermediate Clinical Fellowship to Khandaker (01486/Z/16/Z).

Study Sponsor

The study is jointly sponsored by the Cambridgeshire and Peterborough NHS Foundation Trust and the University of Cambridge.

For peer review only

Competing interests

The authors have no conflicts of interest to declare in relation to this study. The sponsors and funders had no role in the study design or any other aspects relating to the conduct or reporting of the study.

Patient consent

Informed consent will be obtained (see manuscript).

Ethics approval

The study has been approved by the South Central - Oxford B Research Ethics Committee (REC) on 24/04/2018 (Reference: 18/SC/0118).

For peer review only

For peer review only - http://bmjopen.bmj.com/site/about/guidelines.xhtml

Reference

1. Dantzer R, O'Connor JC, Freund GG, Johnson RW, Kelley KW. From inflammation to sickness and depression: when the immune system subjugates the brain. *Nature reviews. Neuroscience* 2008;9(1):46-56.

2. Miller AH, Maletic V, Raison CL. Inflammation and its discontents: the role of cytokines in the pathophysiology of major depression. *Biological psychiatry* 2009;65(9):732-41.

3. Howren MB, Lamkin DM, Suls J. Associations of depression with C-reactive protein, IL-1, and IL-6: a meta-analysis. *Psychosomatic medicine* 2009;71(2):171-86.

4. Haapakoski R, Mathieu J, Ebmeier KP, Alenius H, Kivimaki M. Cumulative meta-analysis of interleukins 6 and 1beta, tumour necrosis factor alpha and C-reactive protein in patients with major depressive disorder. *Brain, behavior, and immunity* 2015;49:206-15.

5. Goldsmith DR, Rapaport MH, Miller BJ. A meta-analysis of blood cytokine network alterations in psychiatric patients: comparisons between schizophrenia, bipolar disorder and depression. *Molecular psychiatry* 2016.

6. Maes M, Bosmans E, De Jongh R, Kenis G, Vandoolaeghe E, Neels H. Increased serum IL-6 and IL-1 receptor antagonist concentrations in major depression and treatment resistant depression. *Cytokine* 1997;9(11):853-8.

7. O'Brien SM, Scully P, Fitzgerald P, Scott LV, Dinan TG. Plasma cytokine profiles in depressed patients who fail to respond to selective serotonin reuptake inhibitor therapy. *Journal of psychiatric research* 2007;41(3-4):326-31.

8. Gimeno D, Kivimaki M, Brunner EJ, Elovainio M, De Vogli R, Steptoe A, et al. Associations of C-reactive protein and interleukin-6 with cognitive symptoms of depression: 12-year follow-up of the Whitehall II study. *Psychological medicine* 2009;39(3):413-23.

For peer review only - http://bmjopen.bmj.com/site/about/guidelines.xhtml

9. Khandaker GM, Pearson RM, Zammit S, Lewis G, Jones PB. Association of serum interleukin 6 and C-reactive protein in childhood with depression and psychosis in young adult life: a population-based longitudinal study. *JAMA psychiatry* 2014;71(10):1121-8.

10. Khandaker GM, Stochl J, Zammit S, Goodyer I, Lewis G, Jones PB. Childhood inflammatory markers and intelligence as predictors of subsequent persistent depressive symptoms: a longitudinal cohort study. *Psychological medicine* 2017:1-12.

11. Zalli A, Jovanova O, Hoogendijk WJ, Tiemeier H, Carvalho LA. Low-grade inflammation predicts persistence of depressive symptoms. *Psychopharmacology* 2016;233(9):1669-78.

12. Davey Smith G, Ebrahim S. 'Mendelian randomization': can genetic epidemiology contribute to understanding environmental determinants of disease? *International journal of epidemiology* 2003;32(1):1-22.

13. Burgess S, Timpson NJ, Ebrahim S, Davey Smith G. Mendelian randomization: where are we now and where are we going? *International journal of epidemiology* 2015;44(2):379-88.

14. Khandaker GM, Zammit S, Burgess S, Lewis G, Jones PB. Association between a functional interleukin 6 receptor genetic variant and risk of depression and psychosis in a population-based birth cohort. *Brain, behavior, and immunity* 2017.

15. Khandaker GM, Dantzer R, Jones PB. Immunopsychiatry: important facts. *Psychological medicine* 2017:1-9.

16. Kappelmann N, Lewis G, Dantzer R, Jones PB, Khandaker GM. Antidepressant activity of anti-cytokine treatment: a systematic review and meta-analysis of clinical trials of chronic inflammatory conditions. *Molecular psychiatry* 2018;23(2):335-43.

17. Raison CL, Rutherford RE, Woolwine BJ, Shuo C, Schettler P, Drake DF, et al. A randomized controlled trial of the tumor necrosis factor antagonist infliximab for

For peer review only - http://bmjopen.bmj.com/site/about/guidelines.xhtml

treatment-resistant depression: the role of baseline inflammatory biomarkers. *JAMA psychiatry* 2013;70(1):31-41.

18. Capuron L, Fornwalt FB, Knight BT, Harvey PD, Ninan PT, Miller AH. Does cytokine-induced depression differ from idiopathic major depression in medically healthy individuals? *Journal of affective disorders* 2009;119(1-3):181-5.
19. Capuron L, Gumnick JF, Musselman DL, Lawson DH, Reemsnyder A, Nemeroff CB, et al. Neurobehavioral effects of interferon-alpha in cancer patients: phenomenology and paroxetine responsiveness of symptom dimensions. *Neuropsychopharmacology : official publication of the American College of Neuropsychopharmacology* 2002;26(5):643-52.
20. Jokela M, Virtanen M, Batty GD, Kivimaki M. Inflammation and Specific Symptoms of Depression. *JAMA psychiatry* 2016;73(1):87-8.
21. Ferrie JE, Kivimaki M, Akbaraly TN, Singh-Manoux A, Miller MA, Gimeno D, et al. Associations between change in sleep duration and inflammation: findings on C-reactive protein and interleukin 6 in the Whitehall II Study. *American journal of epidemiology* 2013;178(6):956-61.
22. Harrison NA, Voon V, Cercignani M, Cooper EA, Pessiglione M, Critchley HD. A Neurocomputational Account of How Inflammation Enhances Sensitivity to Punishments Versus Rewards. *Biological psychiatry* 2016;80(1):73-81.
23. Chu AL, Stochl J, Lewis G, Zammit S, Jones PB, Khandaker GM. Longitudinal Association between Inflammatory Markers and Somatic and Psychological Symptoms of Depression in a Prospective Birth Cohort. 2018.
24. Duivis HE, Vogelzangs N, Kupper N, de Jonge P, Penninx BW. Differential association of somatic and cognitive symptoms of depression and anxiety with inflammation: findings from the Netherlands Study of Depression and Anxiety (NESDA). *Psychoneuroendocrinology* 2013;38(9):1573-85.

For peer review only - http://bmjopen.bmj.com/site/about/guidelines.xhtml

25. Kaser M, Zaman R, Sahakian BJ. Cognition as a treatment target in depression. *Psychological medicine* 2017;47(6):987-89.

26. Sparkman NL, Buchanan JB, Heyen JR, Chen J, Beverly JL, Johnson RW. Interleukin-6 facilitates lipopolysaccharide-induced disruption in working memory and expression of other proinflammatory cytokines in hippocampal neuronal cell layers. *The Journal of neuroscience : the official journal of the Society for Neuroscience* 2006;26(42):10709-16.

27. Donegan JJ, Girotti M, Weinberg MS, Morilak DA. A novel role for brain interleukin-6: facilitation of cognitive flexibility in rat orbitofrontal cortex. *The Journal of neuroscience : the official journal of the Society for Neuroscience* 2014;34(3):953-62.

28. Tang MM, Lin WJ, Pan YQ, Guan XT, Li YC. Hippocampal neurogenesis dysfunction linked to depressive-like behaviors in a neuroinflammation induced model of depression. *Physiology & behavior* 2016;161:166-73.

29. Mackinnon N, Zammit S, Lewis G, Jones PB, Khandaker GM. Association between childhood infection, serum inflammatory markers and intelligence: findings from a population-based prospective birth cohort study. *Epidemiology and infection* 2018;146(2):256-64.

30. Krogh J, Benros ME, Jorgensen MB, Vesterager L, Elfving B, Nordentoft M. The association between depressive symptoms, cognitive function, and inflammation in major depression. *Brain, behavior, and immunity* 2014;35:70-6.

31. Goldsmith DR, Haroon E, Woolwine BJ, Jung MY, Wommack EC, Harvey PD, et al. Inflammatory markers are associated with decreased psychomotor speed in patients with major depressive disorder. *Brain, behavior, and immunity* 2016;56:281-8.

32. Chang HH, Lee IH, Gean PW, Lee SY, Chi MH, Yang YK, et al. Treatment response and cognitive impairment in major depression: association with C-reactive protein. *Brain, behavior, and immunity* 2012;26(1):90-5.

For peer review only - http://bmjopen.bmj.com/site/about/guidelines.xhtml

33. Chan AW, Tetzlaff JM, Altman DG, Laupacis A, Gotzsche PC, Krle AJK, et al. SPIRIT 2013 Statement: defining standard protocol items for clinical trials. *Rev Panam Salud Publica* 2015;38(6):506-14.

34. Calabrese LH, Rose-John S. IL-6 biology: implications for clinical targeting in rheumatic disease. *Nature reviews. Rheumatology* 2014;10(12):720-7.

35. Bonaccorso S, Marino V, Biondi M, Grimaldi F, Ippoliti F, Maes M. Depression induced by treatment with interferon-alpha in patients affected by hepatitis C virus. *Journal of affective disorders* 2002;72(3):237-41.

36. O'Connor JC, Lawson MA, Andre C, Moreau M, Lestage J, Castanon N, et al. Lipopolysaccharide-induced depressive-like behavior is mediated by indoleamine 2,3-dioxygenase activation in mice. *Molecular psychiatry* 2009;14(5):511-22.

37. Haroon E, Raison CL, Miller AH. Psychoneuroimmunology meets neuropsychopharmacology: translational implications of the impact of inflammation on behavior. *Neuropsychopharmacology : official publication of the American College of Neuropsychopharmacology* 2012;37(1):137-62.

38. Khandaker GM, Dantzer R. Is there a role for immune-to-brain communication in schizophrenia? *Psychopharmacology* 2016;233(9):1559-73.

39. Hodes GE, Pfau ML, Leboeuf M, Golden SA, Christoffel DJ, Bregman D, et al. Individual differences in the peripheral immune system promote resilience versus susceptibility to social stress. *Proceedings of the National Academy of Sciences of the United States of America* 2014;111(45):16136-41.

40. WHO. The ICD-10 Classification of Mental and Behavioural Disorder: Clinical Descriptions and Disgnostic Guidelines: World Health Organization, 1992.

41. Beck AT, Steer RA, Brown GK. Beck depression inventory-II. *San Antonio* 1996;78(2):490-8.

For peer review only - http://bmjopen.bmj.com/site/about/guidelines.xhtml

42. Wiles N, Thomas L, Abel A, Barnes M, Carroll F, Ridgway N, et al. Clinical effectiveness and cost-effectiveness of cognitive behavioural therapy as an adjunct to pharmacotherapy for treatment-resistant depression in primary care: the CoBalT randomised controlled trial. *Health technology assessment* 2014;18(31):1-167, vii-viii.

43. Kroenke K, Spitzer RL, Williams JB. The PHQ-9: validity of a brief depression severity measure. *J Gen Intern Med* 2001;16(9):606-13.

44. Woloshin S, Schwartz LM. Distribution of C-reactive protein values in the United States. *The New England journal of medicine* 2005;352(15):1611-3.

45. Woo P, Wilkinson N, Prieur AM, Southwood T, Leone V, Livermore P, et al. Open label phase II trial of single, ascending doses of MRA in Caucasian children with severe systemic juvenile idiopathic arthritis: proof of principle of the efficacy of IL-6 receptor blockade in this type of arthritis and demonstration of prolonged clinical improvement. *Arthritis research & therapy* 2005;7(6):R1281-8.

For peer review only

For peer review only - http://bmjopen.bmj.com/site/about/guidelines.xhtml

Figure legends

Figure 1: Cases of Depression at Age 18 in the ALSPAC Birth Cohort Grouped by Serum IL-6 Levels at Age 9 (Panel A) and by *IL-6R* Genotype (Panel B)

Note: Panel A: Samples of depression were divided by tertiles of interleukin 6 (IL-6) in participants at age 9 years. Cutoff values for the top and bottom thirds of the distribution of IL-6 values in the total sample (cases and noncases combined) were 1.08 and 0.57 pg/mL, respectively. Khandaker et al. JAMA Psychiatry. 2014 Oct;71(10):1121-8. (Reproduced with permission).

Panel B has been adapted from Khandaker et al. Brain Behaviour Immunity. 2018 Mar;69:264-272

Figure 2: Overview of Design and Procedures for the Insight Study

For peer review only

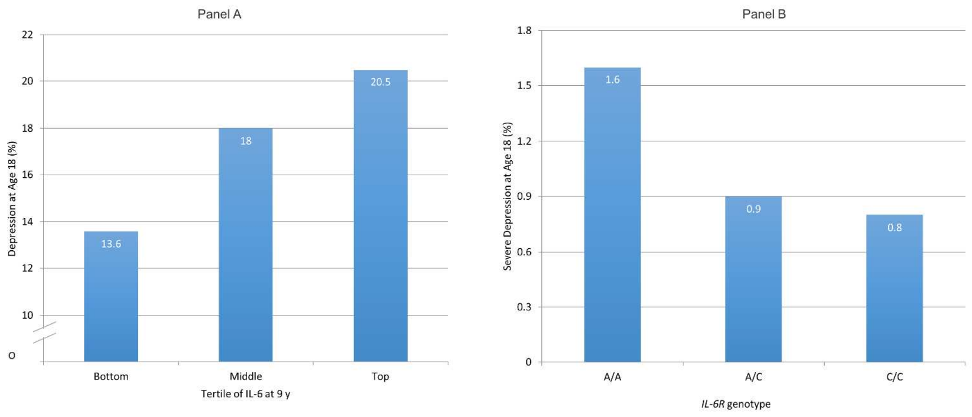

Figure 1

310x143mm (300 x 300 DPI)

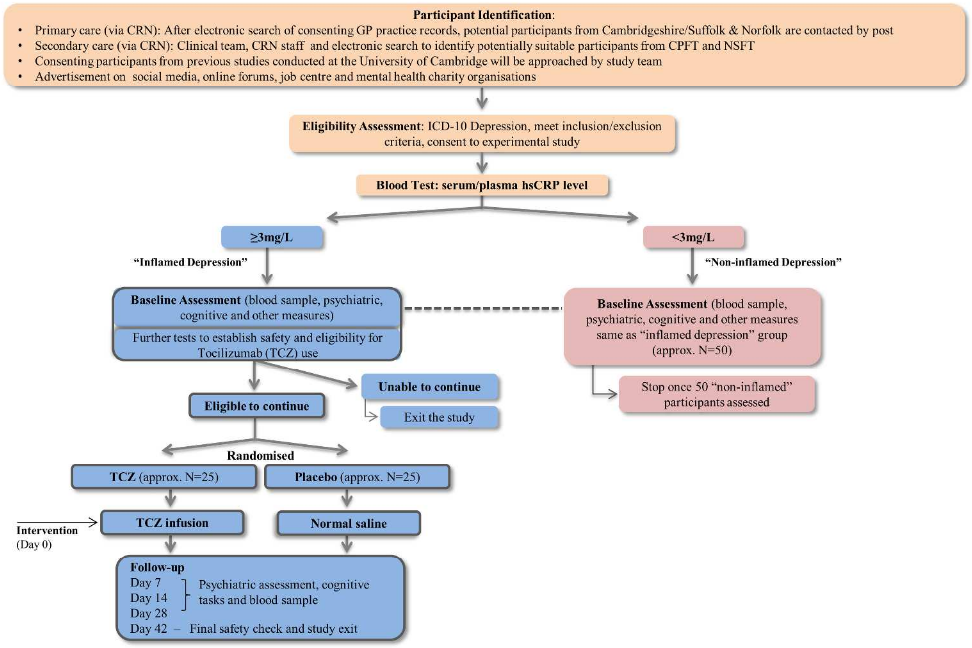

Figure 2

277x182mm (300 x 300 DPI)

STANDARD PROTOCOL ITEMS: RECOMMENDATIONS FOR INTERVENTIONAL TRIALS

Khandaker et al. Protocol for the Insight Study: a randomised controlled trial of single dose tocilizumab in patients with depression and low-grade inflammation

eTable 1: SPIRIT 2013 Checklist: Recommended items to address in a clinical trial protocol and related documents*

Section/item	Item No	Description	Addressed on page number
**Administrative information**			
Title	1	Descriptive title identifying the study design, population, interventions, and, if applicable, trial acronym	1
Trial registration	2a	Trial identifier and registry name. If not yet registered, name of intended registry	3
	2b	All items from the World Health Organization Trial Registration Data Set	3 (ISRCTN1694254)
Protocol version	3	Date and version identifier	-
Funding	4	Sources and types of financial, material, and other support	23
Roles and responsibilities	5a	Names, affiliations, and roles of protocol contributors	1, 23
	5b	Name and contact information for the trial sponsor	23
	5c	Role of study sponsor and funders, if any, in study design; collection, management, analysis, and interpretation of data; writing of the report; and the decision to submit the report for publication, including whether they will have ultimate authority over any of these activities	24

For peer review only - http://bmjopen.bmj.com/site/about/guidelines.xhtml

	5d	Composition, roles, and responsibilities of the coordinating centre, steering committee, endpoint adjudication committee, data management team, and other individuals or groups overseeing the trial, if applicable (see Item 21a for data monitoring committee)	21
**Introduction**			
Background and rationale	6a	Description of research question and justification for undertaking the trial, including summary of relevant studies (published and unpublished) examining benefits and harms for each intervention	5-8
	6b	Explanation for choice of comparators	7-8
Objectives	7	Specific objectives or hypotheses	9-10
Trial design	8	Description of trial design including type of trial (eg, parallel group, crossover, factorial, single group), allocation ratio, and framework (eg, superiority, equivalence, noninferiority, exploratory)	10
**Methods: Participants, interventions, and outcomes**			
Study setting	9	Description of study settings (eg, community clinic, academic hospital) and list of countries where data will be collected. Reference to where list of study sites can be obtained	16
Eligibility criteria	10	Inclusion and exclusion criteria for participants. If applicable, eligibility criteria for study centres and individuals who will perform the interventions (eg, surgeons, psychotherapists)	13, Fig. 2
Interventions	11a	Interventions for each group with sufficient detail to allow replication, including how and when they will be administered	15-16
	11b	Criteria for discontinuing or modifying allocated interventions for a given trial participant (eg, drug dose change in response to harms, participant request, or improving/worsening disease)	18, 20
	11c	Strategies to improve adherence to intervention protocols, and any procedures for monitoring adherence (eg, drug tablet return, laboratory tests)	-
	11d	Relevant concomitant care and interventions that are permitted or prohibited during the trial	-

For peer review only - http://bmjopen.bmj.com/site/about/guidelines.xhtml

Outcomes	12	Primary, secondary, and other outcomes, including the specific measurement variable (eg, systolic blood pressure), analysis metric (eg, change from baseline, final value, time to event), method of aggregation (eg, median, proportion), and time point for each outcome. Explanation of the clinical relevance of chosen efficacy and harm outcomes is strongly recommended	7-8, 16, Table 1
Participant timeline	13	Time schedule of enrolment, interventions (including any run-ins and washouts), assessments, and visits for participants. A schematic diagram is highly recommended (see Figure)	Fig. 2, 15-16
Sample size	14	Estimated number of participants needed to achieve study objectives and how it was determined, including clinical and statistical assumptions supporting any sample size calculations	14
Recruitment	15	Strategies for achieving adequate participant enrolment to reach target sample size	15
**Methods: Assignment of interventions (for controlled trials)**			
Allocation:			
Sequence generation	16a	Method of generating the allocation sequence (eg, computer-generated random numbers), and list of any factors for stratification. To reduce predictability of a random sequence, details of any planned restriction (eg, blocking) should be provided in a separate document that is unavailable to those who enrol participants or assign interventions	14
Allocation concealment mechanism	16b	Mechanism of implementing the allocation sequence (eg, central telephone; sequentially numbered, opaque, sealed envelopes), describing any steps to conceal the sequence until interventions are assigned	14
Implementation	16c	Who will generate the allocation sequence, who will enrol participants, and who will assign participants to interventions	14
Blinding (masking)	17a	Who will be blinded after assignment to interventions (eg, trial participants, care providers, outcome assessors, data analysts), and how	14
	17b	If blinded, circumstances under which unblinding is permissible, and procedure for revealing a participant's allocated intervention during the trial	20
**Methods: Data collection, management, and analysis**			

For peer review only - http://bmjopen.bmj.com/site/about/guidelines.xhtml

Data collection methods	18a	Plans for assessment and collection of outcome, baseline, and other trial data, including any related processes to promote data quality (eg, duplicate measurements, training of assessors) and a description of study instruments (eg, questionnaires, laboratory tests) along with their reliability and validity, if known. Reference to where data collection forms can be found, if not in the protocol	16, Table 1
	18b	Plans to promote participant retention and complete follow-up, including list of any outcome data to be collected for participants who discontinue or deviate from intervention protocols	-
Data management	19	Plans for data entry, coding, security, and storage, including any related processes to promote data quality (eg, double data entry; range checks for data values). Reference to where details of data management procedures can be found, if not in the protocol	21-22
Statistical methods	20a	Statistical methods for analysing primary and secondary outcomes. Reference to where other details of the statistical analysis plan can be found, if not in the protocol	14
	20b	Methods for any additional analyses (eg, subgroup and adjusted analyses)	14
	20c	Definition of analysis population relating to protocol non-adherence (eg, as randomised analysis), and any statistical methods to handle missing data (eg, multiple imputation)	-
**Methods: Monitoring**			
Data monitoring	21a	Composition of data monitoring committee (DMC); summary of its role and reporting structure; statement of whether it is independent from the sponsor and competing interests; and reference to where further details about its charter can be found, if not in the protocol. Alternatively, an explanation of why a DMC is not needed	21
	21b	Description of any interim analyses and stopping guidelines, including who will have access to these interim results and make the final decision to terminate the trial	N/A
Harms	22	Plans for collecting, assessing, reporting, and managing solicited and spontaneously reported adverse events and other unintended effects of trial interventions or trial conduct	20
Auditing	23	Frequency and procedures for auditing trial conduct, if any, and whether the process will be independent from investigators and the sponsor	-
**Ethics and dissemination**			

For peer review only - http://bmjopen.bmj.com/site/about/guidelines.xhtml

Research ethics approval	24	Plans for seeking research ethics committee/institutional review board (REC/IRB) approval	20
Protocol amendments	25	Plans for communicating important protocol modifications (eg, changes to eligibility criteria, outcomes, analyses) to relevant parties (eg, investigators, REC/IRBs, trial participants, trial registries, journals, regulators)	-
Consent or assent	26a	Who will obtain informed consent or assent from potential trial participants or authorised surrogates, and how (see Item 32)	21
	26b	Additional consent provisions for collection and use of participant data and biological specimens in ancillary studies, if applicable	21
Confidentiality	27	How personal information about potential and enrolled participants will be collected, shared, and maintained in order to protect confidentiality before, during, and after the trial	21-22
Declaration of interests	28	Financial and other competing interests for principal investigators for the overall trial and each study site	24
Access to data	29	Statement of who will have access to the final trial dataset, and disclosure of contractual agreements that limit such access for investigators	-
Ancillary and post-trial care	30	Provisions, if any, for ancillary and post-trial care, and for compensation to those who suffer harm from trial participation	-
Dissemination policy	31a	Plans for investigators and sponsor to communicate trial results to participants, healthcare professionals, the public, and other relevant groups (eg, via publication, reporting in results databases, or other data sharing arrangements), including any publication restrictions	22
	31b	Authorship eligibility guidelines and any intended use of professional writers	-
	31c	Plans, if any, for granting public access to the full protocol, participant-level dataset, and statistical code	-
**Appendices**			
Informed consent materials	32	Model consent form and other related documentation given to participants and authorised surrogates	Appendix 1

For peer review only - http://bmjopen.bmj.com/site/about/guidelines.xhtml

Biological specimens	33	Plans for collection, laboratory evaluation, and storage of biological specimens for genetic or molecular analysis in the current trial and for future use in ancillary studies, if applicable	-

*It is strongly recommended that this checklist be read in conjunction with the SPIRIT 2013 Explanation & Elaboration for important clarification on the items. Amendments to the protocol should be tracked and dated. The SPIRIT checklist is copyrighted by the SPIRIT Group under the Creative Commons "Attribution-NonCommercial-NoDerivs 3.0 Unported" license.

For peer review only
