# Supplementary material for: Protocol for the insight study: a randomised controlled trial of single-dose tocilizumab in patients with depression and low-grade inflammation
Source: BMJ Open. 2018 Sep 21;8(9):e025333. doi: 10.1136/bmjopen-2018-025333 (PMC6157523; doi:10.1136/bmjopen-2018-025333)
Supplement: Reviewer comments [file bmjopen-2018-025333.reviewer_comments.pdf]

PEER REVIEW HISTORY

BMJ Open publishes all reviews undertaken for accepted manuscripts. Reviewers are asked to complete a checklist review form (**http://bmjopen.bmj.com/site/about/resources/checklist.pdf**) and are provided with free text boxes to elaborate on their assessment. These free text comments are reproduced below.

ARTICLE DETAILS

**TITLE (PROVISIONAL)**	Protocol for the Insight Study: a randomised controlled trial of single dose tocilizumab in patients with depression and low-grade inflammation
**AUTHORS**	Khandaker, Golam; Oltean, Bianca; Kaser, Muzaffer; Dibben, Claire; Ramana, Rajini; Jadon, Deepak; Dantzer, R; Coles, Alasdair J; Lewis, Glyn; Jones, Peter

This article was not externally reviewed at BMJ Open. Protocols that have been independently assessed prior to submission to BMJ Open are usually fast-tracked to publication on the grounds that further substantial changes will not be possible. This independent assessment will usually be external, independent review for both a substantial grant award from a non-commercial or government funder and ethics approval.
